# Design and Synthesis of Core‐Shell Nanospheres Composed of Heterostructured V_2_O_5_‐CeVO_4_ Toward Efficient Zn‐Ion Storage

**DOI:** 10.1002/advs.202505993

**Published:** 2025-06-11

**Authors:** Dan Liu, Yinxiang Zeng, Yue Xu, Qiang Liu, Deyan Luan, Yan Guo, Xiaojun Gu

**Affiliations:** ^1^ School of Chemistry and Chemical Engineering Inner Mongolia Key Laboratory of Low Carbon Catalysis Inner Mongolia University Hohhot 010021 China; ^2^ Department of Chemistry City University of Hong Kong 83 Tat Chee Avenue Kowloon Hong Kong 999077 China

**Keywords:** aqueous Zn‐ion batteries, cerium vanadate, heterostructures, vanadium oxides

## Abstract

The development of suitable cathode materials is a major scientific challenge for aqueous Zn‐ion batteries (AZIBs). Although vanadium oxides have demonstrated encouraging results, challenges such as sluggish reaction kinetics and severe capacity decay caused by unstable crystal structure and vanadium dissolution still hinder their further application. Herein, Ce‐glycerate nanospheres are used as a self‐engaged template to construct core‐shell nanospheres composed of heterostructured V_2_O_5_‐CeVO_4_ (denoted as VO‐CeVO) by an anion‐exchange strategy. The unique heterostructure with abundant active sites, large specific surface area, and core‐shell design not only boosts ion/electron migration but also inhibits volume change and vanadium dissolution during the repeated Zn^2+^ ion insertion/extraction processes, enabling enhanced Zn^2+^ ion storage performance and structural stability. As expected, the VO‐CeVO cathode delivers a high capacity of 346.3 mAh g^−1^ at 0.5 A g^−1^, excellent rate capability (257.0 mAh g^−1^ at 10 A g^−1^), and outstanding cycling performance (over 10 000 cycles).

## Graphical abstract

Core‐shell nanospheres composed of heterostructured V_2_O_5_‐CeVO_4_ are constructed as the cathode for high‐performance aqueous Zn‐ion batteries. The unique heterostructure and core‐shell structure can facilitate ion/electron migration and mitigate volume change. Additionally, the introduction of CeVO_4_ is able to inhibit V dissolution and stabilize V_2_O_5_, thereby reinforcing the structural stability. As expected, the V_2_O_5_‐CeVO_4_ cathode delivers excellent rate capability and cycling performance.

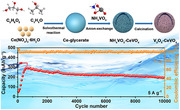

## 1 Introduction

Rechargeable aqueous Zn‐ion batteries (AZIBs) have received extensive attention in the field of large‐scale energy storage due to their merits of low cost, safety, and environmental friendliness.^[^ ^1^, ^2^ ^]^ Zn metal possesses earth abundance, low redox potential, and high theoretical capacity (820 mAh g^−1^).^[^ ^3^, ^4^, ^5^ ^]^ The selection of suitable cathode materials is essential for improving the energy density and cycle life, which is crucial for thedevelopment of AZIBs.^[^ ^6^, ^7^ ^]^ Based on the open framework, unique structure, and multivalent states of vanadium, vanadium oxides can undergo multiple redox reactions from V^2+^ to V^5+^, providing abundant active sites for Zn^2+^ storage.^[^ ^8^, ^9^ ^]^ However, the application of vanadium oxides in AZIBs is mainly hindered by the sluggish reaction kinetics and unstable crystal structure. The repeated insertion/extraction of Zn^2+^ ions in vanadium oxides induces significant structural changes and lattice stress, leading to serious structural damage and dissolution of vanadium during prolonged cycling.^[^ ^10^, ^11^ ^]^

Numerous strategies have been employed to address these issues and improve the Zn^2+^ ion storage performance of vanadium oxides, such as pillaring the interlayers by the pre‐intercalation of guest species (e.g., metal ions, structured water, or polymer molecules), creating structural defects, and compositing with other materials.^[^ ^12^ ^]^ Proper pillaring in the layered structure can expand the interlayer spacing and stabilize the crystal structure, thereby improving the cycling stability.^[^ ^13^ ^]^ However, the presence of guest species in the active sites inhibits the insertion of Zn^2+^ ions, leading to the loss of specific capacity.^[^ ^14^ ^]^ Coupling vanadium oxides with other materials to form composites is considered as a promising way to enhance electrochemical properties.^[^ ^15^, ^16^ ^]^ Compared with simple mixing, constructing heterostructures enables a tighter binding of the two phases and generates a built‐in electric field at their interfaces, which facilitates rapid charge transfer and ion diffusion.^[^ ^17^, ^19^ ^]^ Rare earth elements with unique 4f electronic structures possess special optical, electrical, and magnetic properties, making them suitable for fabricating energy storage materials and optimizing electron transfer efficiency.^[^ ^20^, ^21^, ^22^ ^]^ In particular, cerium vanadate (CeVO_4_) is abundant in nature with excellent chemical stability and electrochemical activity, enabling its application in AZIBs.^[^ ^23^, ^24^, ^25^ ^]^ In addition, designing distinctive nanostructures has become an efficient strategy for electrode materials to boost the electrochemical performance.^[^ ^26^ ^]^ Specially, the core‐shell structure with a high surface area increases the electrode‐electrolyte contact area and provides numerous electrochemical active sites to accelerate ion diffusion.^[^ ^27^, ^28^ ^]^ More importantly, the interior of the core‐shell structure can provide additional void space to alleviate the volume change and mechanical strain caused by the repeated insertion/extraction of Zn^2+^ ions.^[^ ^29^ ^]^ Metal‐glycerate has been reported as an ideal precursor or sacrificial template for the design of micro/nanomaterials with porous, hollow, and core‐shell structures for capacitors and batteries due to its tunable morphology and composition.^[^ ^26^, ^30^ ^]^

Based on the above considerations, herein a facile anion‐exchange strategy followed by a thermal annealing treatment is used to synthesize core‐shell nanospheres consisting of V_2_O_5_‐CeVO_4_ (denoted as VO‐CeVO) heterostructures using the Ce‐glycerate as the self‐engaged template. The as‐prepared VO‐CeVO nanospheres with core‐shell structures offer abundant active sites and large specific surface area. Moreover, the interfacial engineering of the heterojunction can not only improve the electronic conductivity and ion diffusion coefficient of the cathode but also effectively mitigate vanadium dissolution, contributing to improved rate capability and structural stability. Benefiting from the multiple structural and compositional advantages, the VO‐CeVO cathode exhibits a high capacity of 257.0 mAh g^−1^ at a current density of 10 A g^−1^ and prolonged cycling performance over 10 000 cycles at 5 A g^−1^ with a retained capacity of 193.6 mAh g^−1^.

## 2 Results and Discussion

**Figure 1:**
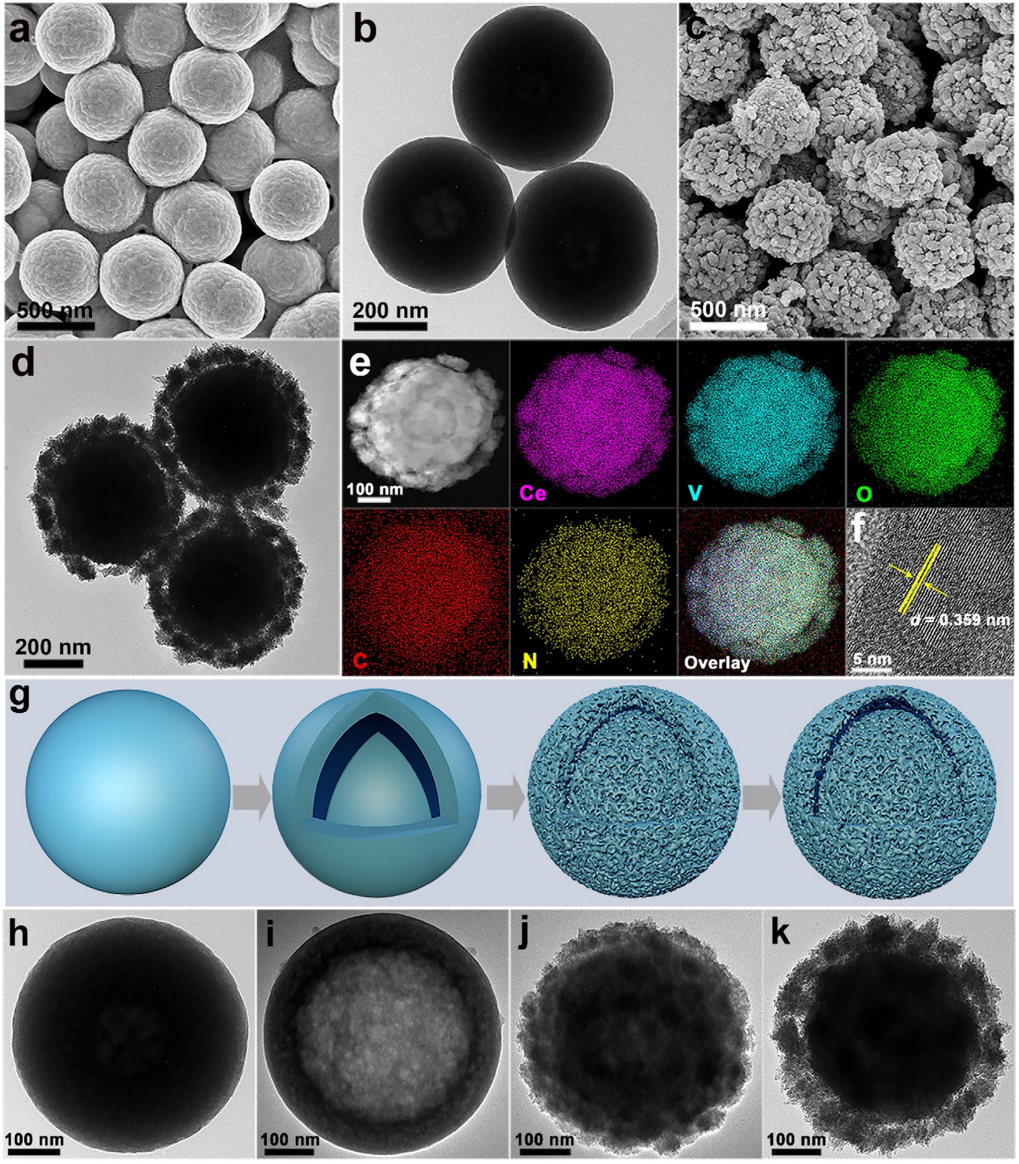
a) FESEM and b) TEM images of Ce‐glycerate. c) FESEM image, d) TEM image, e) HAADF‐STEM and the corresponding elemental mapping images, and f) HRTEM image of NH_4_VO_3_‐CeVO_4_. g) Schematic diagram illustrating the time‐dependent morphology evolution of the NH_4_VO_3_‐CeVO_4_ intermediates. h–k) TEM images of the NH_4_VO_3_‐CeVO_4_ intermediates with various anion‐exchange time: h) 0, i) 10, j) 60, and k) 120 min.

Ce‐glycerate nanospheres prepared via a solvothermal approach are used as the self‐engaged templates. Field‐emission scanning electron microscopy (FESEM) images (**Figure** **1**a; Figure S1a, Supporting Information) display that the Ce‐glycerate nanospheres are well dispersed and uniform with an average diameter of ≈540 nm. Transmission electron microscopy (TEM) images further indicate that the Ce‐glycerate nanospheres have a solid interior and a rather smooth surface (Figure 1b; Figure S1b, Supporting Information). X‐ray diffraction (XRD) pattern of Ce‐glycerate shows a weak peak at ≈23°, suggesting its amorphous nature (Figure S2, Supporting Information). Afterward, Ce‐glycerate nanospheres undergo an anion‐exchange process to harvest the NH_4_VO_3_‐CeVO_4_ intermediate. While the original spherical shape is maintained, the surface becomes rough, and a shell layer of ≈50 nm forms on its exterior (Figure 1c,d; Figure S3, Supporting Information). The XRD pattern of NH_4_VO_3_‐CeVO_4_ (Figure S4, Supporting Information) confirms the existence of NH_4_VO_3_ (JCPDS No. 076–0191) and CeVO_4_ (JCPDS No. 079–1065). The high‐angle annular dark‐field scanning transmission electron microscopy (HAADF‐STEM), elemental mapping images, and energy‐dispersive X‐ray (EDX) linear scanning results of an individual NH_4_VO_3_‐CeVO_4_ nanosphere (Figure 1e; Figure S5, Supporting Information) reveal the uniform distribution of C, Ce, N, O, and V elements. The EDX spectrum of NH_4_VO_3_‐CeVO_4_ (Figure S6, Supporting Information) further validates the above results. From the high‐resolution TEM (HRTEM) image of NH_4_VO_3_‐CeVO_4_ (Figure 1f), lattice fringes with an interplanar spacing of 0.359 nm can be observed, corresponding to the (021) plane of NH_4_VO_3_. The selected area electron diffraction (SAED) pattern of NH_4_VO_3_‐CeVO_4_ (Figure S7, Supporting Information) shows two sets of diffraction rings attributed to the (001) crystal plane of NH_4_VO_3_ and the (200) and (112) planes of CeVO_4_, which further verifies the coexistence of both phases.

Furthermore, the formation process of the core‐shell structure and the microstructural changes of the intermediates were studied by adjusting the anion‐exchange time (0, 10, 60, and 120 min), as illustrated in Figure 1g–k. The Ce‐glycerate solid nanospheres (Figure 1h) are converted into hollow nanospheres with a smooth surface (Figure 1i) after the anion‐exchange process for 10 min, which is mainly caused by the difference in diffusion rates between the inward‐moving VO_3_^−^ ions and outward‐migrating Ce^3+^ ions.^[^ ^31^, ^32^ ^]^ With the increase in reaction time, VO_3_^−^ ions gradually diffuse into the interior, leading to the growth of nanoparticles both on the surface and interior of the nanospheres (Figure 1j). As the anion‐exchange time prolongs to 120 min, a well‐defined gap forms between the exterior and interior of the nanospheres, creating the core‐shell nanospheres (Figure 1k). XRD patterns of the intermediates with different anion‐exchange time (Figure S8, Supporting Information) indicate the presence of NH_4_VO_3_ (JCPDS No. 076–0191) and CeVO_4_ (JCPDS No. 079–1065). The peak intensity increases gradually with the extension of anion‐exchange time, proving the improvement of crystallinity. Moreover, the HAADF‐STEM, elemental mapping images, and corresponding EDX linear scanning results of the intermediates (Figure S9, Supporting Information) further confirm the uniform distribution of Ce, V, O, C, and N elements. As detected by inductively coupled plasma‐optical emission spectrometer (ICP‐OES) and organic element analyzer (Table S1, Supporting Information), there is a gradual increase in the contents of V, Ce, and N, accompanied by a decrease in the C content, with the increment of anion‐exchange time.

**Figure 2:**
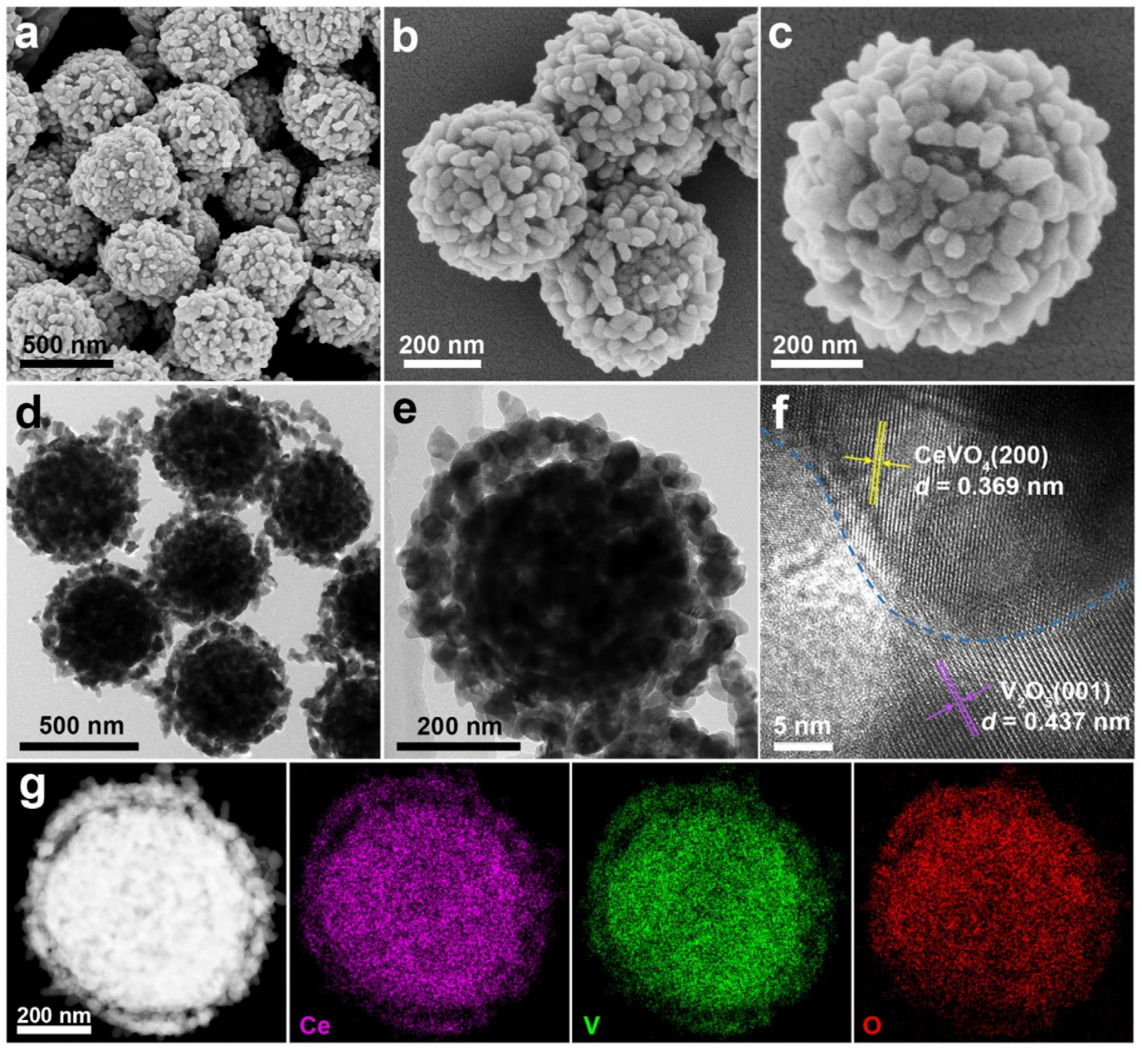
a–c) FESEM images, d,e) TEM images, f) HRTEM image, g) HAADF‐STEM and elemental mapping images of VO‐CeVO.

**Figure 3:**
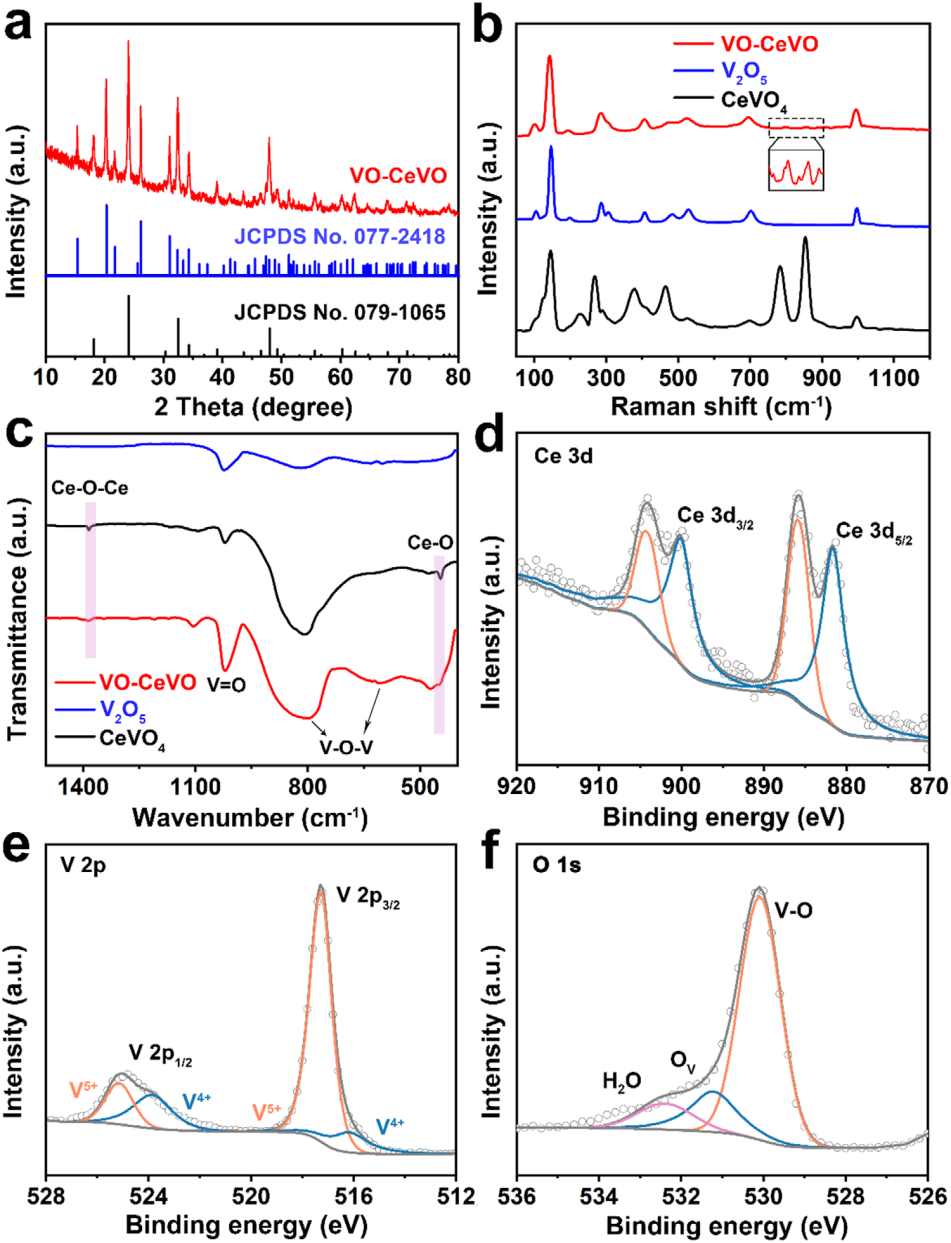
a) XRD pattern of VO‐CeVO. b) Raman spectra and c) FT‐IR spectra of VO‐CeVO, V_2_O_5_, and CeVO_4_. High‐resolution d) Ce 3d, e) V 2p, and f) O 1s XPS spectra of VO‐CeVO. O_v_ represents oxygen vacancies in f).

After annealing in air at 400 °C for 2 h, the optimized NH_4_VO_3_‐CeVO_4_ intermediate transforms into VO‐CeVO, which displays a nanosphere structure assembled from interconnected nanoparticles (**Figure** **2**a–c; Figure S10a, Supporting Information). TEM images of VO‐CeVO verify the well‐maintained core‐shell nanospheres (Figure 2d,e; Figure S10b, Supporting Information). From the HRTEM image (Figure 2f, Figure S10c, Supporting Information), the lattice spacings of 0.369 and 0.437 nm are attributed to the (200) plane of CeVO_4_ and the (001) plane of V_2_O_5_, respectively, revealing the formation of heterointerface, which is indicated by the blue line. As shown in the HAADF‐STEM, elemental mapping images, and EDX linear scanning results (Figure 2g; Figure S11, Supporting Information), Ce, V, and O elements are distributed homogeneously throughout an individual VO‐CeVO nanosphere, thus CeVO_4_ and V_2_O_5_ are homogeneously distributed throughout the core‐shell structure. EDX spectrum of VO‐CeVO further corroborates these findings (Figure S12, Supporting Information) and the content of Ce is ≈14.1% as detected by ICP‐OES (Table S2, Supporting Information). Furthermore, the XRD pattern of VO‐CeVO shows the characteristic peaks of V_2_O_5_ (JCPDS No. 077–2418) and CeVO_4_ (JCPDS No. 079–1065), demonstrating the formation of VO‐CeVO composites (**Figure** **3**a), which is confirmed by the SAED pattern (Figure S13, Supporting Information). For comparison, pure V_2_O_5_ and CeVO_4_ were also synthesized, as confirmed by the XRD patterns, FESEM, and TEM images (Figures S14–S16, Supporting Information). The Raman spectra of VO‐CeVO, V_2_O_5_, and CeVO_4_ are shown in Figure 3b, in which the peaks located at 142.3 and 284.8 cm^−1^ can be attributed to the bending vibration of the V─O and V═O bonds, respectively.^[^ ^33^ ^]^ The peaks at 853.9 and 783.7 cm^−1^ correspond to the internal symmetric stretching mode *ν*_1_ (A_1_g) and asymmetric stretching mode *ν*_3_ (Eg) of CeVO_4_, respectively.^[^ ^24^ ^]^ In the Fourier transform infrared (FT‐IR) spectra of VO‐CeVO, V_2_O_5_, and CeVO_4_ (Figure 3c), the peaks located at 598 and 794 cm^−1^ are assigned to the symmetric and asymmetric stretching vibrations of the V─O─V bond, and the peak at 1012 cm^−1^ belongs to the stretching vibration of the V═O bond.^[^ ^33^, ^34^ ^]^ The peaks at 1383 and 468 cm^−1^ correspond to the stretching vibrations of the Ce─O─Ce and Ce─O bonds, respectively.^[^ ^24^, ^35^ ^]^ The above results validate the successful synthesis of the VO‐CeVO heterostructure.

X‐ray photoelectron spectroscopy (XPS) was further adopted to identify the elemental composition and valence states of VO‐CeVO. The XPS survey spectrum confirms the existence of Ce, V, and O elements in VO‐CeVO (Figure S17, Supporting Information). In the high‐resolution Ce 3d XPS spectrum (Figure 3d), the peaks located at 881.7 and 885.8 eV are assigned to Ce 3d_5/2_, whereas the peaks at 900.1 and 904.1 eV correspond to Ce 3d_3/2_, suggesting the existence of Ce^3+^.^[^ ^36^, ^37^ ^]^ The high‐resolution V 2p spectrum (Figure 3e) displays two pairs of peaks attributed to V^5+^ (517.3 and 525.1 eV) and V^4+^ (516.2 and 523.9 eV).^[^ ^38^ ^]^ Figure 3f shows the high‐resolution O 1s XPS spectrum, where three peaks located at 530.1, 531.2, and 532.4 eV belong to the V─O bond, oxygen vacancy, and structural water, respectively.^[^ ^15^, ^33^ ^]^ The presence of oxygen vacancies in VO‐CeVO is further verified by electron paramagnetic resonance (EPR) measurements (Figure S18, Supporting Information). Nitrogen sorption‐desorption isotherm measurements indicate that VO‐CeVO owns a higher Brunauer‐Emmett‐Teller (BET) surface area of 43.7 m^2^ g^−1^ compared to V_2_O_5_ (5.9 m^2^ g^−1^) and CeVO_4_ (16.7 m^2^ g^−1^) (Figure S19, Supporting Information).

**Figure 4:**
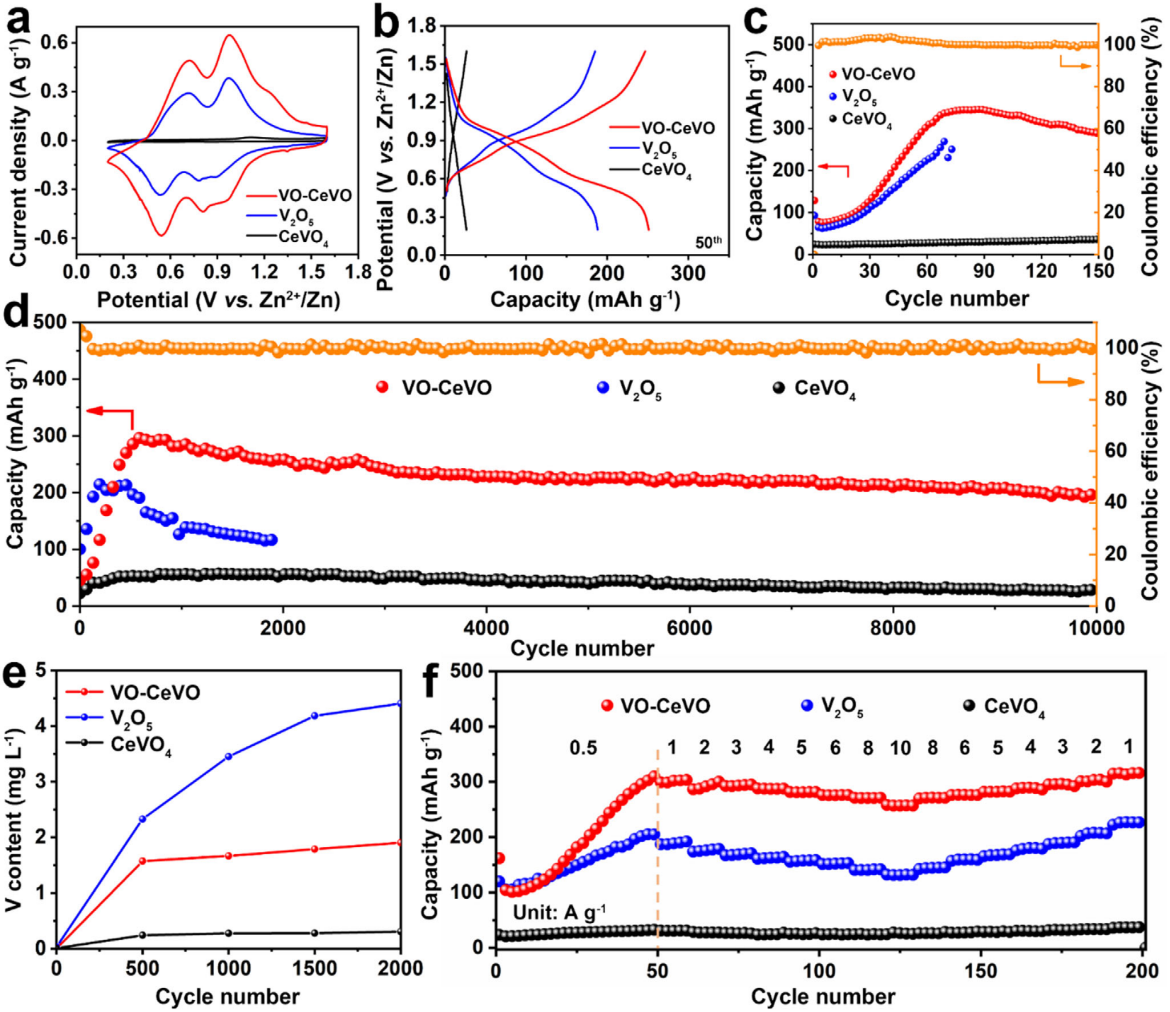
a) CV curves at 0.2 mV s^−1^, b) GCD curves, and c) the corresponding cycling performance at 0.5 A g^−1^, d) cycling performance at 5 A g^−1^, e) mass content of the dissolved vanadium in the electrolyte during the cycling test, and f) rate performance of the VO‐CeVO, V_2_O_5_, and CeVO_4_ electrodes.

To evaluate the electrochemical performance of the VO‐CeVO cathode, a coin‐type battery was assembled with Zn foil as the anode. The cyclic voltammogram (CV) curves of VO‐CeVO, V_2_O_5_, and CeVO_4_ are tested at 0.2 mV s^−1^ (**Figure** **4**a). The VO‐CeVO cathode exhibits a much higher response current than V_2_O_5_ and CeVO_4_, revealing that the VO‐CeVO heterostructure can remarkably increase the electrochemical activity and capacity. There are two pairs of redox peaks at 0.72/0.55 and 0.97/0.81 V, suggesting a two‐step reversible Zn^2+^ insertion/extraction process.^[^ ^39^ ^]^ Figure 4b,c shows the galvanostatic charge‐discharge (GCD) curves and the corresponding cycling performance of the VO‐CeVO, V_2_O_5_, and CeVO_4_ cathodes at 0.5 A g^−1^. Two distinct discharge/charge stages represent a two‐step reaction process, which is consistent with the CV curves. The VO‐CeVO cathode demonstrates a maximum discharge capacity of 346.3 mAh g^−1^, retaining a capacity of 289.4 mAh g^−1^ after 150 cycles. Even at a high current density of 5 A g^−1^, the VO‐CeVO cathode delivers a high capacity of 193.6 mAh g^−1^ after 10 000 cycles (Figure 4d), significantly outperforming V_2_O_5_ (115.8 mAh g^−1^ after 1890 cycles) and CeVO_4_ (28.0 mAh g^−1^ after 10 000 cycles). The VO‐CeVO cathode requires 70 and 574 cycles to reach the maximum capacity at 0.5 and 5 A g^−1^, respectively (Figure 4c,d). The activation process is influenced by the current density, as higher current density will increase the electrode polarization, thus narrowing the activation voltage window and delaying the activation process.^[^ ^40^, ^41^ ^]^ To gain insight into vanadium dissolution during the cycling process, the concentration of dissolved vanadium for these cathodes in electrolytes was measured by ICP‐OES (Figure 4e). For the VO‐CeVO electrode, the concentration of vanadium in the electrolyte is much smaller than that of V_2_O_5_ after 2000 cycles, indicating that the synergistic effect of the VO‐CeVO heterostructure can effectively enhance structural stability.^[^ ^18^ ^]^ Figure 4f shows the rate performance of the VO‐CeVO, V_2_O_5_, and CeVO_4_ cathodes, which is measured after activation at 0.5 A g^−1^ for 50 cycles, and the initial discharge capacities of VO‐CeVO and V_2_O_5_ are 161.8 and 119.8 mAh g^−1^, respectively. At the current densities of 1, 2, 3, 4, 5, 6, 8, and 10 A g^−1^, the average discharge capacities of VO‐CeVO are 300.0, 293.5, 293.5, 287.4, 281.2, 275.8, 270.8, and 257.0 mAh g^−1^, respectively. When the current density returns to 1 A g^−1^, the discharge capacity recovers to 315.5 mAh g^−1^, which is much higher than that of V_2_O_5_ and CeVO_4_. All these results reveal that the VO‐CeVO cathode demonstrates the most comprehensive electrochemical performance compared to V_2_O_5_, CeVO_4_, and other previously reported vanadium‐based cathodes (Table S3, Supporting Information).

**Figure 5:**
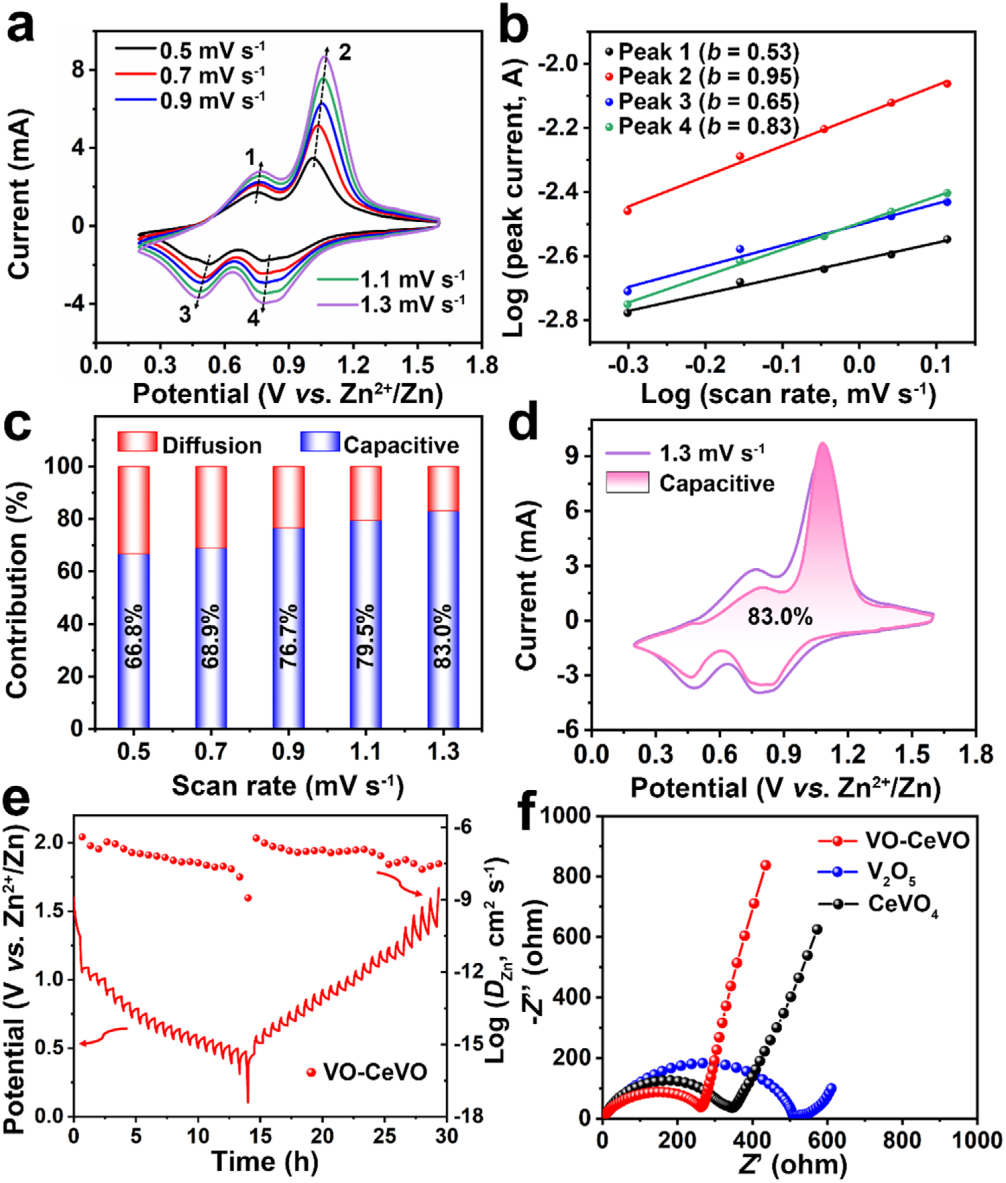
a) CV curves at different scan rates, b) determination of the *b* values using the relationship between peak currents and scan rates, c) capacitive contribution, d) CV curve at 1.3 mV s^−1^, e) discharge and charge GITT curve and the corresponding *D*_Zn_ of the VO‐CeVO electrode. f) Nyquist plots of the VO‐CeVO, V_2_O_5_, and CeVO_4_ electrodes.

To further investigate the charge storage kinetics of the VO‐CeVO electrode, the CV, electrochemical impedance spectroscopy (EIS), and galvanostatic intermittent titration technique (GITT) were performed. **Figure** **5**a shows the CV curves of the VO‐CeVO cathode at different scanning rates. There are two pairs of redox peaks, suggesting a two‐step reversible Zn^2+^ insertion/extraction process.^[^ ^39^, ^42^ ^]^ Peaks 1 and 3 correspond to the reversible V^4+^/V^3+^ transition, while peaks 2 and 4 are associated with the reversible V^5+^/V^4+^ transition.^[^ ^42^, ^43^, ^44^ ^]^ As the scan rate increases from 0.5 to 1.3 mV s^−1^, the shape of the CV curves remains consistent, indicating the good reversibility of VO‐CeVO. The relationship between peak current (*i*) and sweep rate (*v*) can be described as: *i* = *av^b^*, where *a* and *b* are tunable parameters.^[^ ^45^ ^]^ As shown in Figure 5b, the calculated *b* values for the four peaks are 0.53, 0.95, 0.65, and 0.83, respectively, reflecting that the reaction is governed by both capacitive and diffusion‐controlled processes. Peaks 1 and 3 are primarily diffusion‐controlled, while peaks 2 and 4 are mainly capacitive‐controlled, which may be caused by the faster Zn^2+^ diffusion kinetics during the initial V^5+^/V^4+^ redox stage compared to the subsequent V^4+^/V^3+^ transition.^[^ ^44^ ^]^ In addition, the calculated capacitive contribution to the total capacity increases gradually with the scan rate (Figure 5c), contributing to the superior rate performance of VO‐CeVO.^[^ ^46^ ^]^ At a scan rate of 1.3 mV s^−1^, the capacitive contribution reaches 83.0% of the total capacity (Figure 5d). Furthermore, the diffusion coefficient of Zn^2+^ (*D*_Zn_) for the VO‐CeVO electrode was evaluated by GITT test. The *D*_Zn_ of VO‐CeVO is ≈10^−9^–10^−6^ cm^2^ s^−1^, much higher than that of V_2_O_5_ and CeVO_4_ (Figure 5e; Figure S20, Supporting Information), which aligns with the enhanced rate performance. The EIS plots in Figure 5f further reveal that the charge transfer resistance of VO‐CeVO is lower than that of V_2_O_5_ and CeVO_4_, demonstrating that the synergistic effect of the heterostructure and core‐shell nanospheres enhances charge transfer kinetics. To further analyze the migration of Zn^2+^, the density functional theory (DFT) calculations were performed. The binding energy of Zn atom on the VO‐CeVO interface (−0.8 eV) is between that of V_2_O_5_ (−1.53 eV) and CeVO_4_ (1.54 eV), indicating that it is favorable for both Zn^2+^ adsorption and desorption (Figure S21, Supporting Information).^[^ ^34^, ^47^ ^]^ In addition, the lower diffusion energy barriers of Zn^2+^ in both VO‐CeVO interface and interlayer compared to V_2_O_5_ suggest that the VO‐CeVO can facilitate Zn^2+^ migration. In addition, the Zn^2+^ diffusion energy barrier at the VO‐CeVO interface is even lower than that in the VO‐CeVO interlayer, indicating that the VO‐CeVO interface plays a more prominent role in Zn^2+^ migration (Figure S22, Supporting Information).^[^ ^48^ ^]^

**Figure 6:**
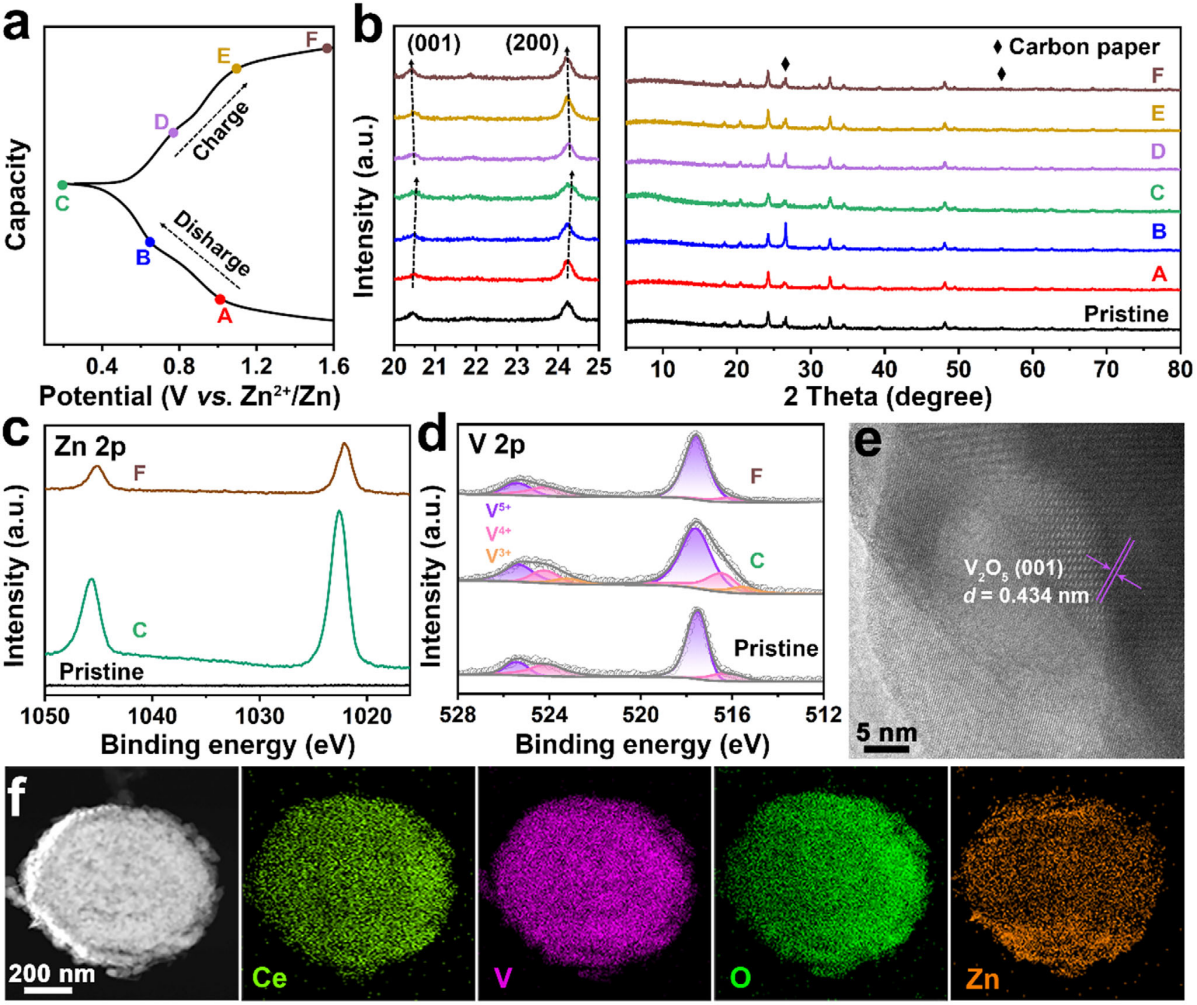
Structural evolution of the VO‐CeVO electrode during the discharge/charge processes. a) GCD curve at 0.5 A g^−1^ and the corresponding ex situ b) XRD patterns, high‐resolution c) Zn 2p and d) V 2p XPS spectra. e) HRTEM image, f) HAADF‐STEM image, and the corresponding elemental mapping images at the fully discharged state.

The electrochemical mechanism was further explored by analyzing the VO‐CeVO electrode at different charge/discharge states via ex situ XRD, XPS, FESEM, and TEM. From the GCD curve at 0.5 A g^−1^ and the corresponding ex situ XRD patterns (**Figure** **6**a,b), it is evident that no new peaks appear during the charge/discharge processes, while the (001) peak of V_2_O_5_ and the (200) peak of CeVO_4_ shift toward higher angles upon discharging and revert to their initial positions at the fully charged state, verifying the reversible (de)intercalation of Zn^2+^ ions.^[^ ^13^ ^]^ To further study the valence state variation of the VO‐CeVO electrode during the Zn^2+^ ion storage process, ex situ XPS characterization was performed. As shown in Figure 6c, there is no Zn 2p signal at the pristine state, while two strong peaks corresponding to Zn 2p_1/2_ (1045.6 eV) and Zn 2p_3/2_ (1022.6 eV) can be observed at the fully discharged state (point C), confirming the successful intercalation of Zn^2+^ ions. After fully charging to 1.6 V (point F), the Zn 2p signal weakens due to the Zn^2+^ extraction.^[^ ^49^ ^]^ In the high‐resolution V 2p XPS spectra (Figure 6d), the intensity of V^4+^ increases and new peaks (515.6 and 523.2 eV) ascribed to V^3+^ appear at the fully discharged state.^[^ ^50^, ^51^ ^]^ During the discharge process, V^5+^ is predominantly reduced to V^4+^, while only a small fraction of V^4+^ undergoes further reduction to V^3+^. Thus, the capacity contribution of the VO‐CeVO electrode mainly originates from peaks 2 and 4 corresponding to the V^5+^/V^4+^ redox reaction, whereas peaks 1 and 3 associated with the V^4+^/V^3+^ transition contribute only marginally.^[^ ^44^ ^]^ When charged to 1.6 V, the V 2p XPS spectrum reverts to its initial state and the V^3+^ signaldisappears. The HRTEM image of VO‐CeVO at the fully discharged state (Figure 6e) reveals a slight contraction of the (001) crystal plane for V_2_O_5_ due to the strong electrostatic interaction after Zn^2+^ ion insertion. The FESEM, TEM, HAADF‐STEM, and corresponding elemental mapping images of VO‐CeVO at the fully discharged state (Figure 6f; Figure S23, Supporting Information) show that the core‐shell nanospheres remain intact without the appearance of lamellar alkaline by‐products. The uniform distribution of Ce, V, O, and Zn elements further confirms the intercalation of Zn^2+^ ions, in accordance with the above results. When charged to 1.6 V, VO‐CeVO maintains the nanosphere structure and the lattice spacing of (001) crystal plane for V_2_O_5_ can recover to 0.439 nm (Figure S24, Supporting Information). Moreover, the Zn^2+^ ions might also undergo reversible (de)intercalation into CeVO_4_ as verified by the ex situ XRD, HAADF‐STEM image, and the corresponding elemental mapping images (Figure S25, Supporting Information).

To verify the feasibility of the practical application of the Zn||VO‐CeVO battery, pouch batteries were further assembled (Figure S26, Supporting Information). The Zn||VO‐CeVO pouch battery can provide a maximum capacity of 249.9 mAh g^−1^ at 5 A g^−1^ and maintain a high capacity of 237.8 mAh g^−1^ after 2000 cycles, which is much better than Zn||V_2_O_5_ and Zn||CeVO_4_. Furthermore, two Zn||VO‐CeVO pouch batteries can light up the light‐emitting diode consisting of 36 bulbs with the ZIB logo, suggesting the potential applicability in energy storage devices.

## 3 Conclusion

In summary, we have developed an anion‐exchange strategy to construct core‐shell nanospheres with VO‐CeVO heterostructures by using Ce‐glycerate nanospheres as the template. The heterostructure design induces a built‐in electric field at the two‐phase interfaces, which promotes charge transfer and ion diffusion, thus enhancing the rate capability of the VO‐CeVO electrode. Moreover, the core‐shell nanosphere structure can offer sufficient active sites and alleviate volume change during the repeated Zn^2+^ ion insertion/extraction processes. Benefiting from the desired structure and composition, the obtained VO‐CeVO cathode exhibits outstanding electrochemical performance for AZIBs, achieving stable cycling over 10000 cycles at 5 A g^−1^ with a high reversible capacity of 193.6 mAh g^−1^, outperforming the V_2_O_5_ and CeVO_4_ counterparts. This work provides a feasible strategy for the design and construction of high‐performance vanadium oxide cathodes for AZIBs.

## Conflict of Interest

The authors declare no conflict of interest.

## Supporting information



Supporting Information

## Data Availability

The data that support the findings of this study are available from the corresponding author upon reasonable request.
