# Supplementary material for: Design and Synthesis of Core‐Shell Nanospheres Composed of Heterostructured V2O5‐CeVO4 Toward Efficient Zn‐Ion Storage
Source: Adv Sci (Weinh). 2025 Jun 11;12(34):e05993. doi: 10.1002/advs.202505993 (PMC12442618; doi:10.1002/advs.202505993)
Supplement: Supplementary file 1 — Supporting Information [file ADVS-12-e05993-s001.docx]

Supporting Information

**Experimental Section**

*Synthesis of Ce-glycerate nanospheres.* 0.25 mmol of Ce(NO_3_)_3_·6H_2_O was dispersed into 20 mL of isopropanol by ultrasound. Then, 4 mL of glycerol was added into the above solution under stirring for 30 min to form a colorless transparent solution. Then, the obtained mixture was transferred into a Teflon-lined stainless-steel autoclave and heated at 180 °C for 6 h. After the reactor is cooled to room temperature, the Ce-glycerate nanospheres powder was collected after being rinsed with ethanol and dried at 60 °C for 12 h.

*Synthesis of* *NH_4_VO_3_-CeVO_4_* *nanospheres.* 20 mg of the obtained Ce-glycerate nanospheres was dispersed into 40 mL of absolute ethanol under ultrasound. Then, 10 mL of NH_4_VO_3_ (0.04 mol L^-1^) aqueous solution was added into the above mixture solution under stirring at 70 °C for 2 h. The NH_4_VO_3_-CeVO_4_ nanospheres were harvested after being rinsed with ethanol several times and dried at 60 °C for 12 h.

*Synthesis of V_2_O_5_-CeVO_4_ (denoted as VO-CeVO) nanospheres.* The as-synthesized NH_4_VO_3_-CeVO_4_ was annealed at 400 °C for 2 h in air with a heating rate of 5 °C min^-1^ to obtain VO-CeVO.

*Synthesis of* *CeVO_4_ nanospheres.* For comparison, CeVO_4_ was synthesized by a similar strategy as V_2_O_5_-CeVO_4_ but with the decreased amount of NH_4_VO_3_ (10 mL, 0.02 mol L^-1^) aqueous solution.

*Synthesis of V_2_O_5_.* For comparison, V_2_O_5_ was synthesized by annealing commercial NH_4_VO_3_ at 400 °C for 2 h in air with a heating rate of 5 °C min^-1^.

*Materials characterization.* The crystalline phase of the samples was characterized by X-ray diffraction (XRD, D8 ADVANCE). The transmission electron microscopy (TEM, JEM-2010, JEOL) with energy dispersive X-ray (EDX) spectroscopy attachment and field-emission scanning electron microscopy (FESEM, Regulus 8100) were adopted to examine the morphology and composition of the materials. X-ray photoelectron spectroscopy (XPS, ESCALABXI+) was employed to analyze the chemical compositions and elemental valances. Raman analysis was recorded on a RENISHAW inVia Microscope Raman microscope with a laser excitation source of 532 nm. Fourier transform infrared (FT-IR) spectroscopy was recorded under the wavenumber of 400-4000 cm^-1^ by an infrared spectrophotometer (Thermo Fisher Scientific, NEXUS-670). Nitrogen sorption-desorption isotherm measurement (Autosorb-IQ2, Micromeritics) was applied to investigate the Brunauer-Emmett-Teller (BET) surface area and pore size distribution of the samples. Electron paramagnetic resonance (EPR, Bruker EMXplus) spectra were used to study the oxygen vacancies. Inductively coupled plasma-optical emission spectrometer (ICP-OES, iCAP PRO) was used to determine the contents of V and Ce elements in NH_4_VO_3_-CeVO_4_. Organic element analyzer (OEA, UNICUBE) was used to measure the contents of C and N elements.

*Electrochemical measurements.* The slurry was prepared by mixing active material (70 wt%), acetylene black (20 wt%) and polyvinylidene fluoride (PVDF, 10 wt%) in N-methyl-2-pyrrolidone (NMP) solvent with stirring and then spread onto carbon paper to make the cathode with the loading mass of about 1.0 mg cm^-2^. Afterwards, the cathode was dried in a vacuum oven at 70 ^o^C for 24 h. The CR2430 type coin cells were assembled in air to investigate the electrochemical performance of the cathode materials with Zn(CF_3_SO_3_)_2_ aqueous solution (3 mol L^-1^), glass fiber membrane, and Zn foil as the electrolyte, separator, and anode, respectively. The amount of added electrolyte in each coin cell was about 0.3 mL and the thickness of Zn foil was about 0.2 mm. To further verify the performance of the Zn||VO-CeVO battery at high loading mass, pouch batteries were assembled with about 20 mg of active material loaded on a 16 cm^2^ carbon paper. The galvanostatic charge-discharge (GCD) tests were conducted with the LAND (CT3001A) battery test system in a voltage range of 0.2-1.6 V. Cyclic voltammetry (CV) and electrochemical impedance spectroscopy (EIS) measurements were executed on the electrochemical workstation (CHI660E). The theoretical capacity of the electrode is calculated with the following equation:^[1]^

$$\text{C}\text{ = }\frac{\text{n}\text{F}}{\text{3600}\left( M_{w}/1000 \right)}$$

where *n*, F and *M*_w_ is the number of electrons participating in the redox reaction, Faraday constant (96485 C mol^-1^) and the molar mass of the cathode material, respectively. Based on the above equation, the calculated theoretical capacities of V_2_O_5_ and CeVO_4_ are 589 and 210.2 mAh g^-1^, respectively. As detected by ICP-OES, the mass contents of Ce and V in VO-CeVO are 14.1% and 48.4%, respectively. Thus, the mass contents of CeVO_4_ and V_2_O_5_ in VO-CeVO are 25.7% and 74.3%, respectively, and the theoretical capacity of VO-CeVO calculated based on the ratio of CeVO_4_ to V_2_O_5_ is about 491.6 mAh g^-1^. For galvanostatic intermittent titration technique (GITT) measurement, the battery was discharged/charged at 100 mA g^-1^ for 10 min, followed by a 30 min relaxation process to reach the voltage equilibrium. The Zn^2+^ diffusion coefficient (*D*_Zn_, cm^2^ s^-1^) was calculated by the following equation:^[2]^

$$\text{D}_{\text{Zn}}\text{ = }\frac{\text{4}}{\text{π}\text{τ}}\left( \frac{\text{m}_{\text{B}}\text{V}_{\text{M}}}{\text{M}_{\text{B}}\text{S}} \right)^{\text{2}}\left( \frac{{\text{∆}\text{E}}_{\text{s}}}{{\text{∆}\text{E}}_{\text{t}}} \right)^{\text{2}}$$

$$\text{ }\text{= }\frac{\text{4}}{\text{π}\text{τ}}\text{L}^{\text{2}}\left( \frac{{\text{∆}\text{E}}_{\text{s}}}{{\text{∆}\text{E}}_{\text{t}}} \right)^{\text{2}}$$

Where $\text{τ}$ (s) is the relaxation time of the current pulse. *L* (cm) is the Zn^2+^ ion diffusion length, which is close to the thickness of coated slurry. ${\text{∆}\text{E}}_{\text{s}}$ (V) is the voltage change between two adjacent equilibrium states, and $\text{∆E}_{\text{t}}$ (V) is the voltage change caused by charging and discharging at constant current.

*Density functional theory (DFT) calculation methods.* The DFT calculation was performed by the Vienna Ab initio Simulation package (VASP),^[3]^ and the exchange-correlation energy was approximately described by Perdew, Burke, and Ernzerhof (PBE) functional based on the generalized gradient approximation (GGA).^[4]^ The plane wave basis sets with projector-augmented wave (PAW) pseudopotentials were applied in structure optimization.^[5]^ The plane-wave cutoff energy was set to 400 eV. The convergence tolerance of geometry optimization was set as 1.0 × 10^−5^ eV for energy, and all the forces on each atom were smaller than 0.05 eV/Å. For the configuration optimizations and electronic structure calculation, the GGA+U method was adopted to describe the Ce 4f and V 3d electrons, and an effective U parameter was 3.1 eV.^[6]^ The heterostructure consists of 2 CeVO_4_ layers and 2 V_2_O_5_ layer, yielding a thickness of 18.73 Å. The thickness of the vacuum layer vertical to the slab is 10.47 Å. All the structures discussed in this study were visualized in the Visualization for Electronic and Structural Analysis (VESTA).

The binding energy (*E*_b_) of Zn atom on slab was calculated by:

*E*_b_ = *E*_slab+Zn_ – *E*_slab_ – *E*_Zn_

Where *E*_slab+Zn_, *E*_slab_, and *E*_Zn_ are the total energy of the slab adsorbed with Zn atom, the energy of slab and Zn atom, respectively.

**
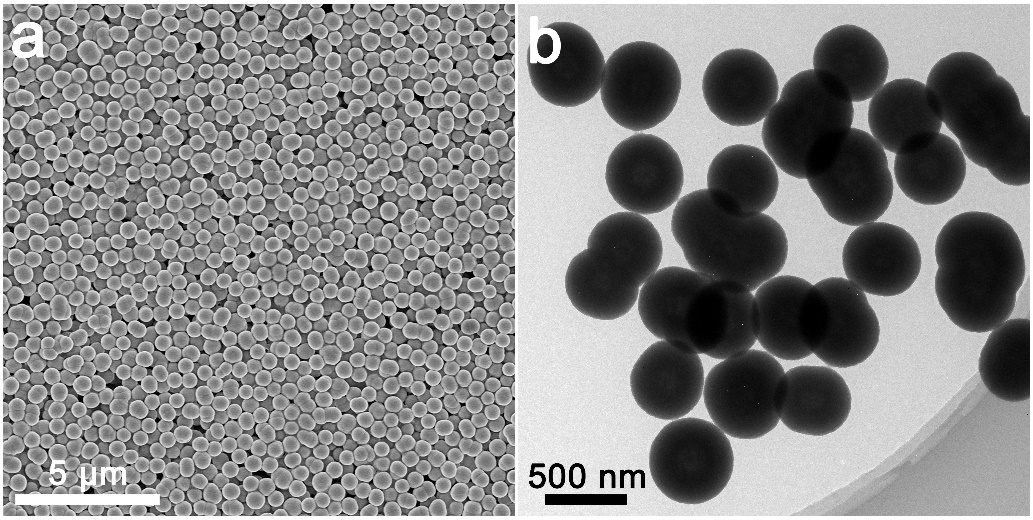
**

**Figure S1.** (a) FESEM and (b) TEM images of Ce-glycerate nanospheres.





**Figure S2.** XRD pattern of Ce-glycerate nanospheres.


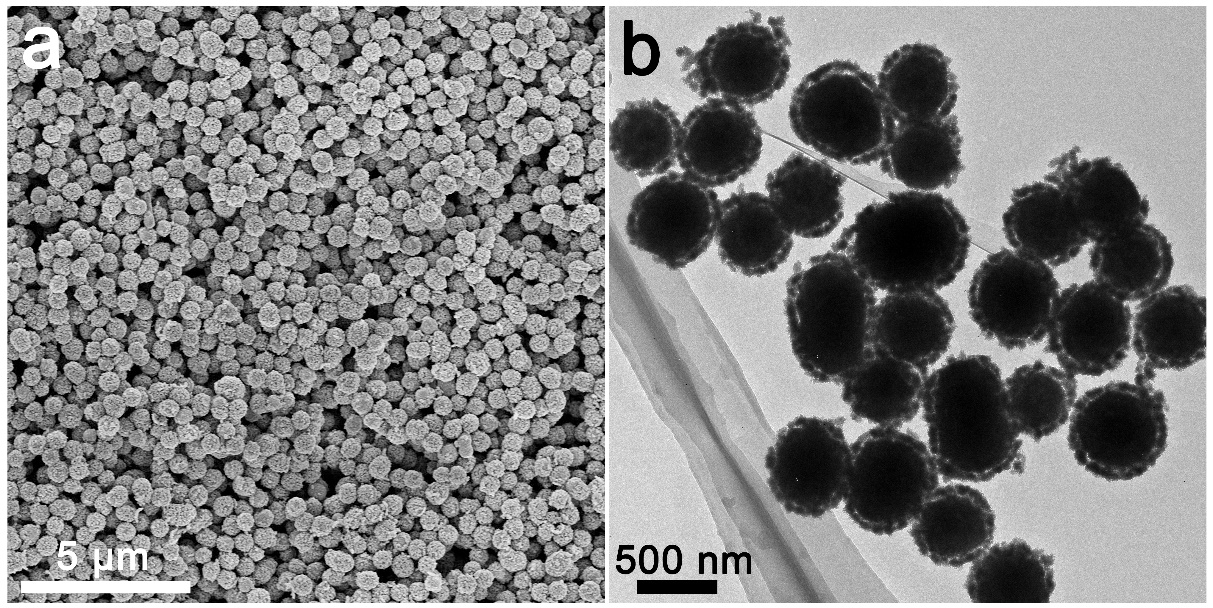


**Figure S3.** (a) FESEM and (b) TEM images of NH_4_VO_3_-CeVO_4_.





**Figure S4.** XRD pattern of NH_4_VO_3_-CeVO_4_.

**
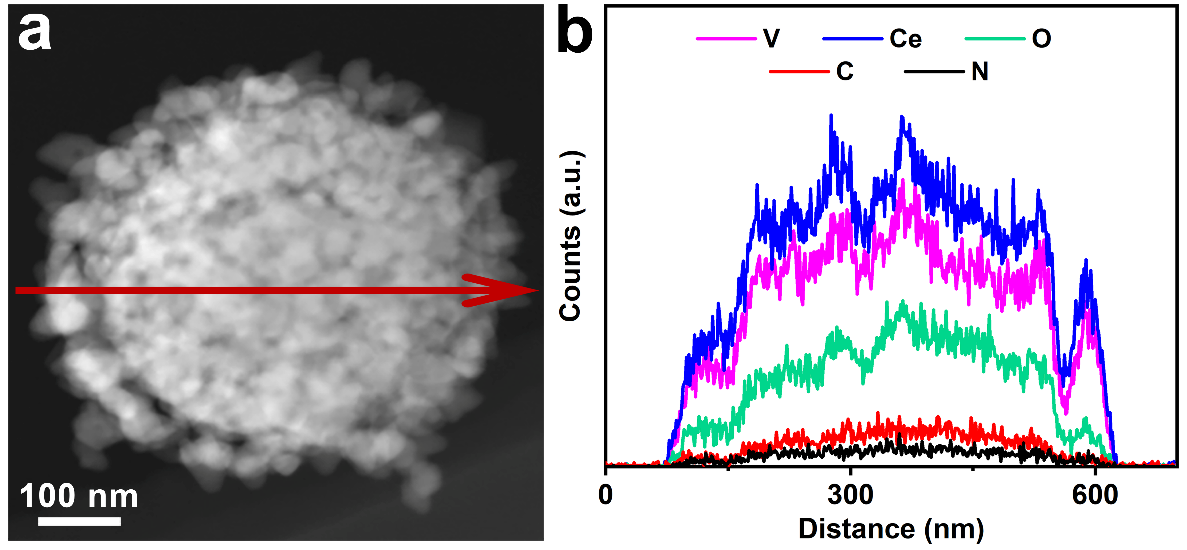
**

**Figure S5.** (a) HAADF-STEM image and (b) the corresponding EDX linear element distribution along the red arrow in (a) of an individual NH_4_VO_3_-CeVO_4_ nanosphere.

**

**

**Figure S6.** EDX spectrum of NH_4_VO_3_-CeVO_4_.


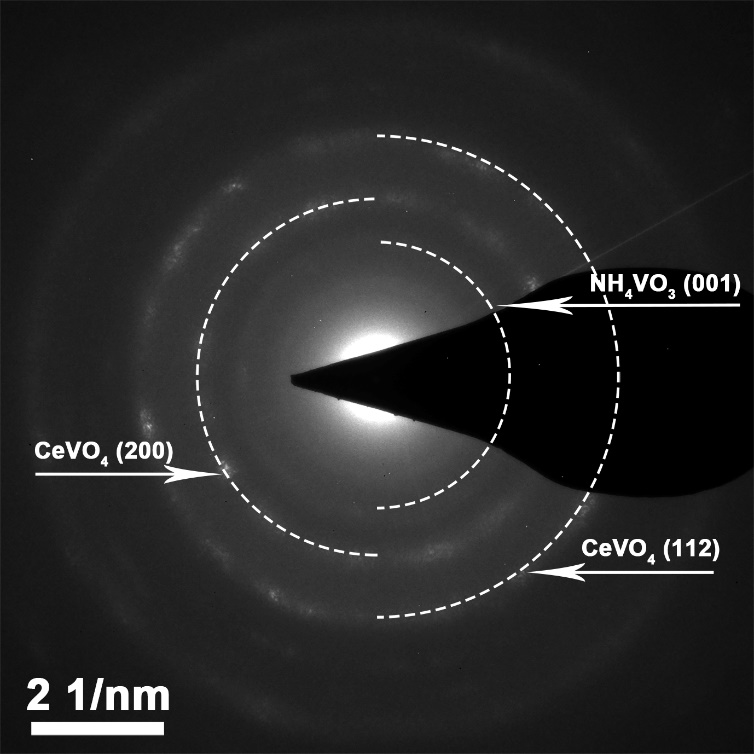


**Figure S7.** SAED pattern of NH_4_VO_3_-CeVO_4_.





**Figure S8.** XRD patterns of NH_4_VO_3_-CeVO_4_ with different anion-exchange time.


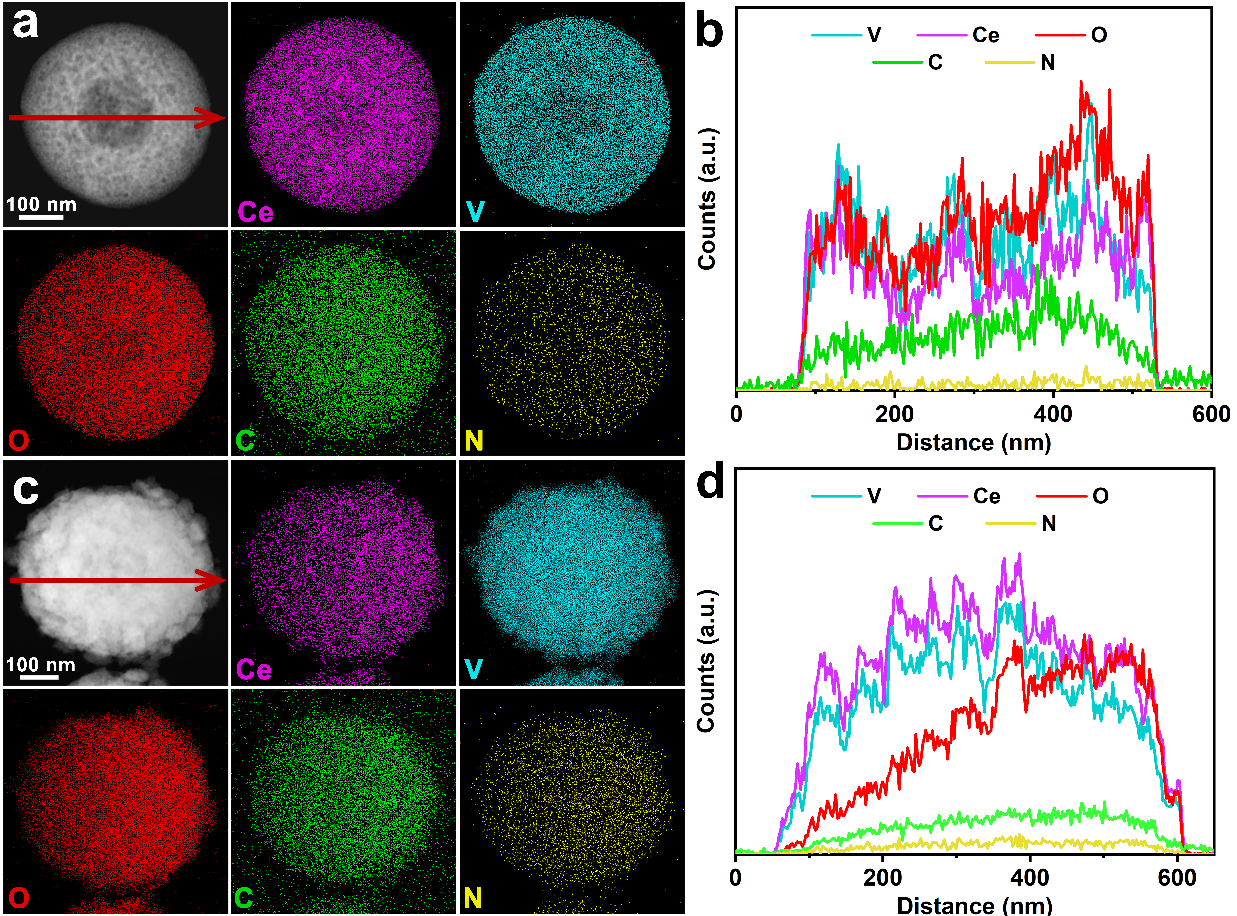


**Figure S9.** (a,c) HAADF-STEM and the corresponding elemental mapping images, and (b,d) the corresponding EDX linear elemental distributions along the red arrow in (a,c) of an individual NH_4_VO_3_-CeVO_4_ nanosphere with anion-exchange time of (a,b) 10 min and (c,d) 60 min.


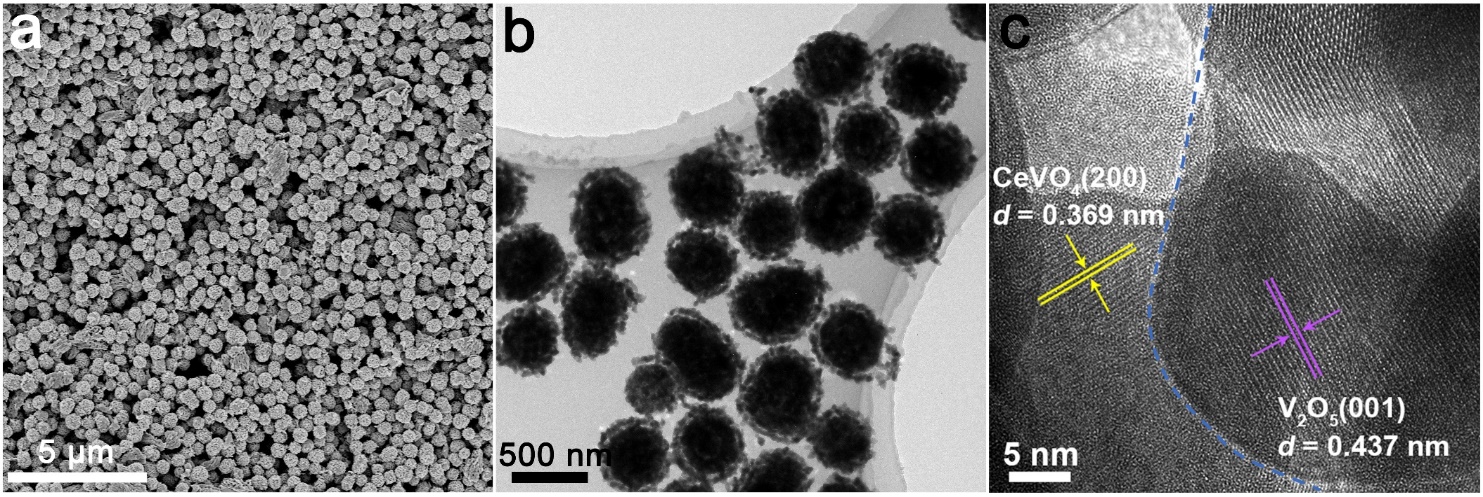


**Figure S10.** (a) FESEM and (b) TEM and (c) HRTEM images of VO-CeVO.


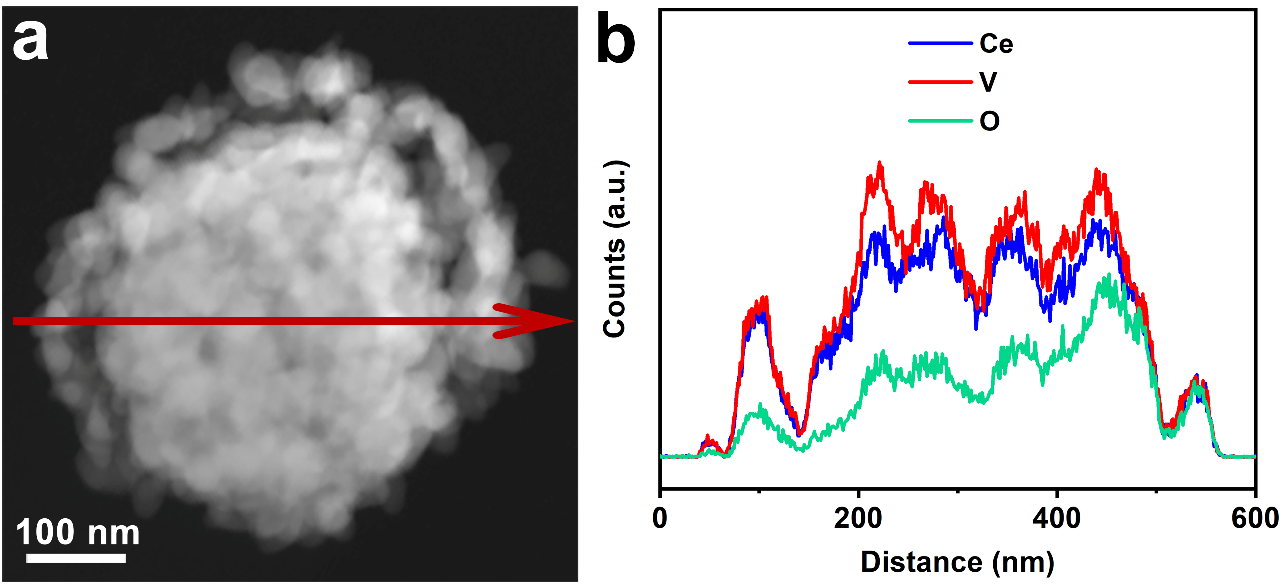


**Figure S11.** (a) HAADF-STEM image and (b) the corresponding EDX linear elemental distributions along the red arrow in (a) of an individual VO-CeVO nanosphere.





**Figure S12.** EDX spectrum of VO-CeVO.


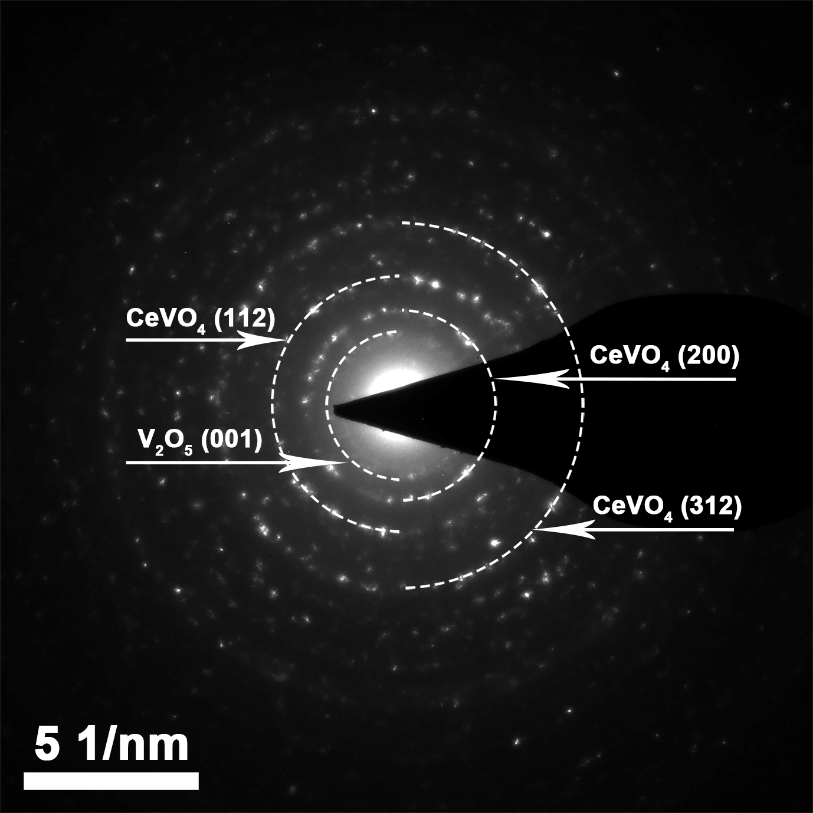


**Figure S13.** SAED pattern of VO-CeVO.


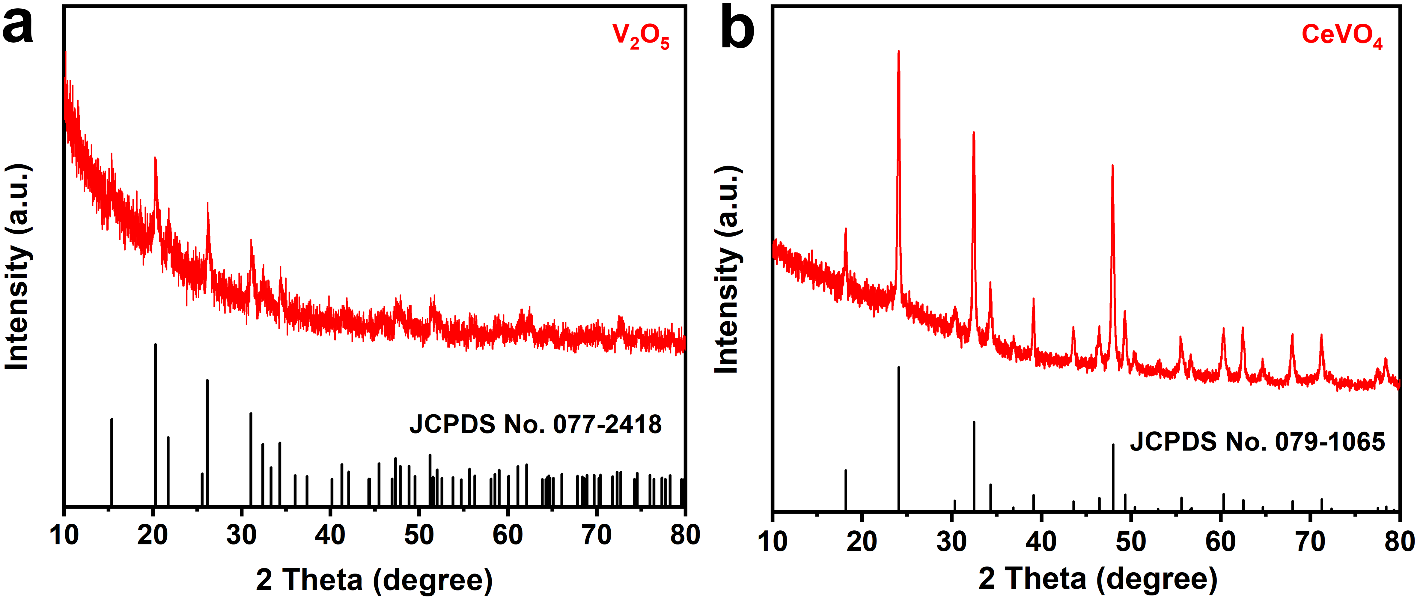


**Figure S14.** XRD patterns of (a) V_2_O_5_ and (b) CeVO_4_.


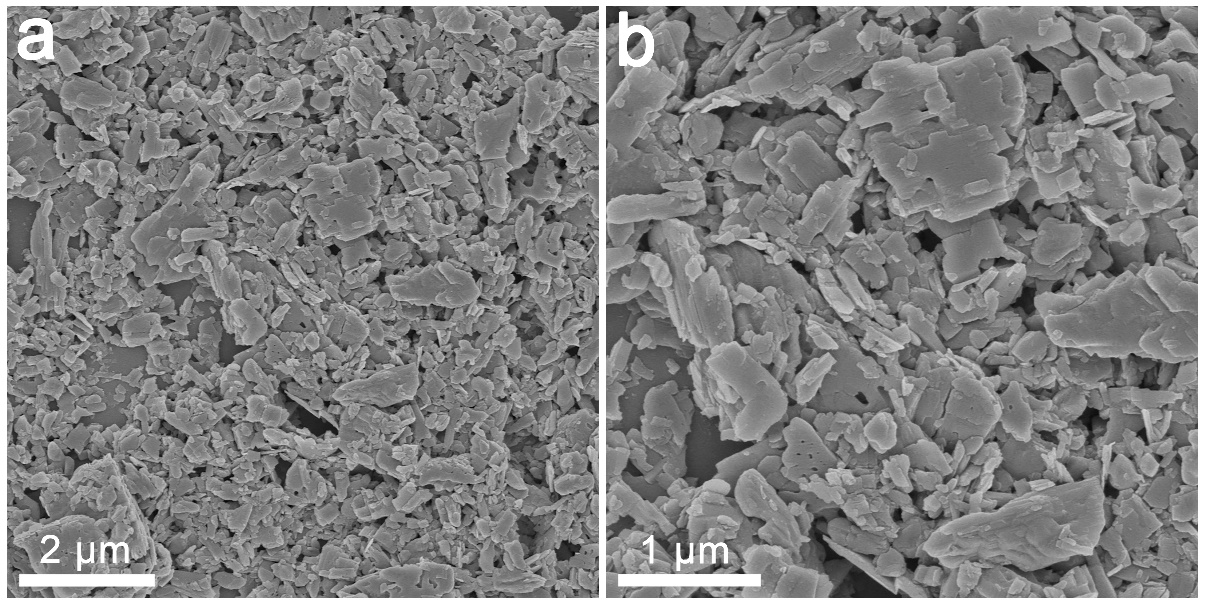


**Figure S15.** FESEM images of V_2_O_5_.

**
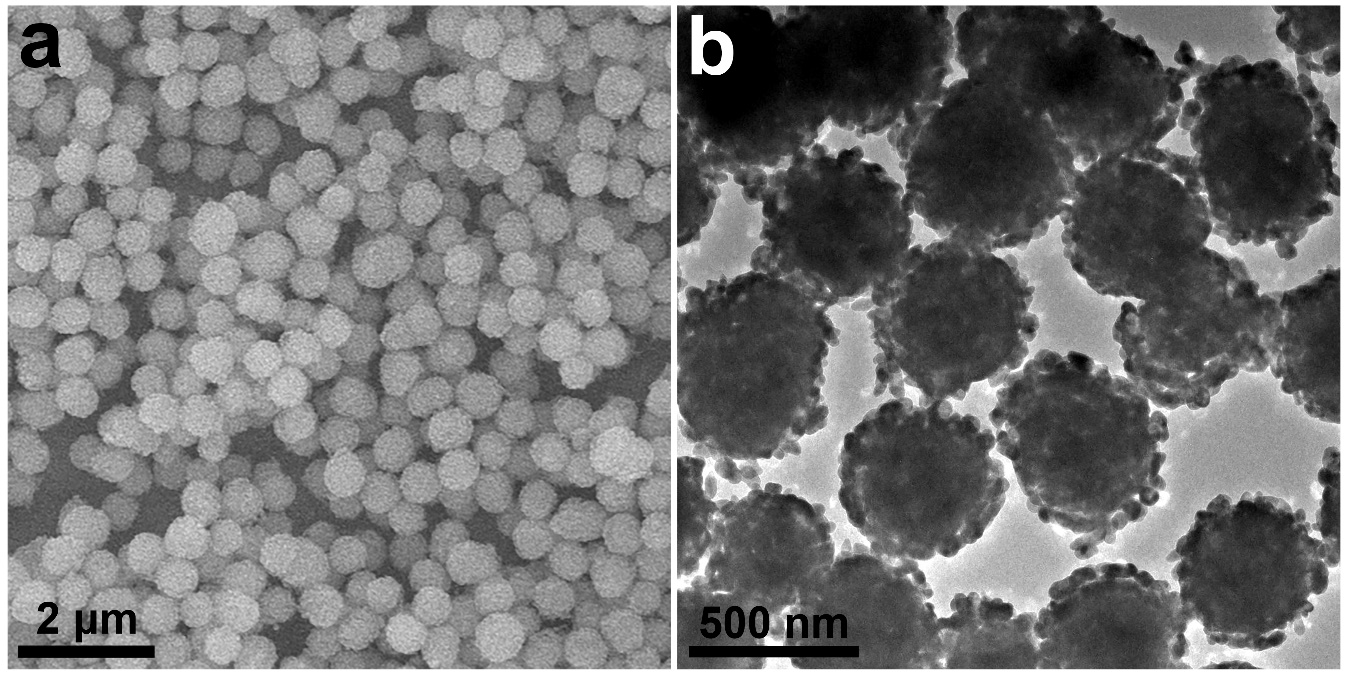
**

**Figure S16.** (a) FESEM and (b) TEM images of CeVO_4_.

**

**

**Figure S17.** XPS survey spectrum of VO-CeVO.





**Figure S18.** EPR curves of VO-CeVO, V_2_O_5_, and CeVO_4_ samples.

**
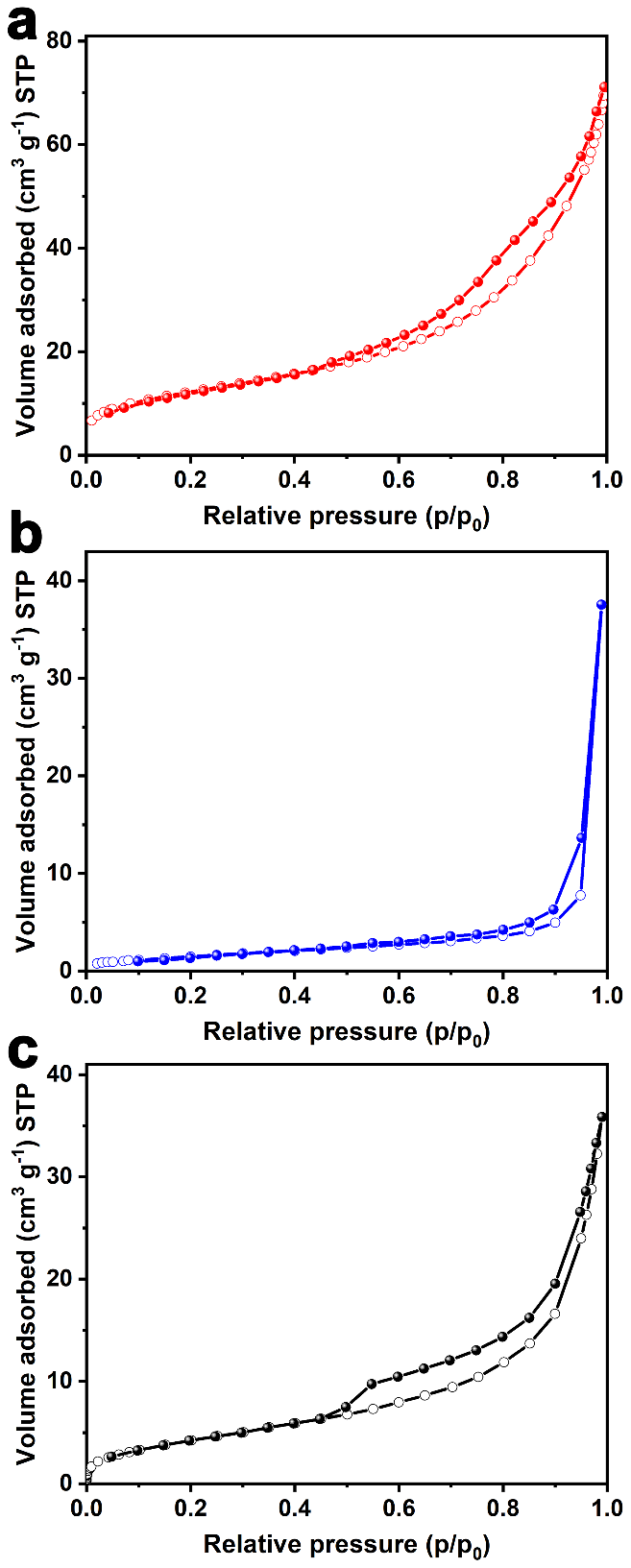
**

**Figure S19.** Nitrogen adsorption-desorption isotherm curves of (a) VO-CeVO, (b) V_2_O_5_, and (c) CeVO_4_.


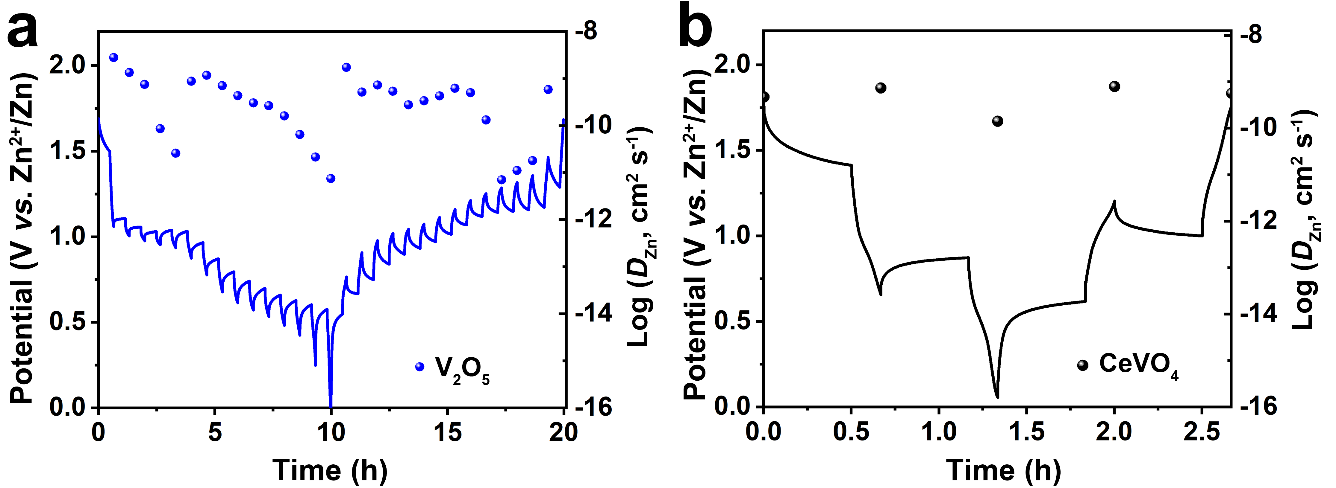


**Figure S20.** Discharge and charge GITT curves and the corresponding *D*_Zn_ of (a) V_2_O_5_ and (b) CeVO_4_ electrodes.


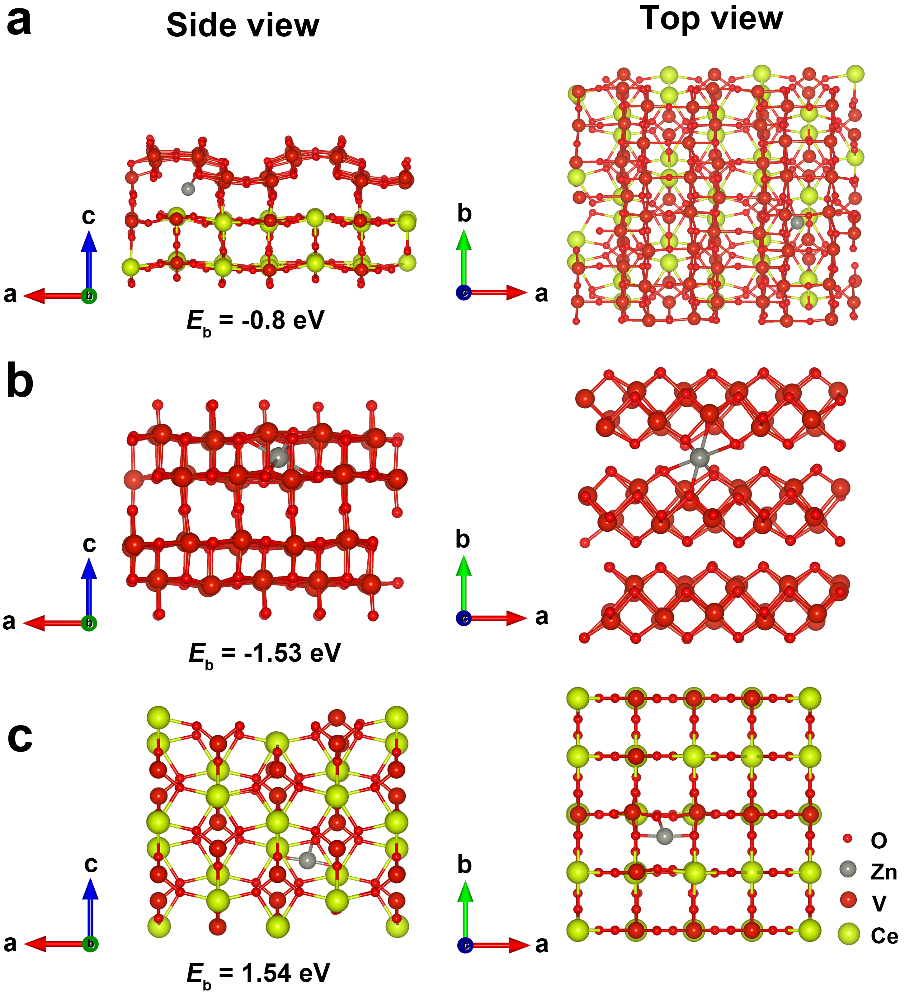


**Figure S21.** The optimized structures of (a) VO-CeVO interface, (b) V_2_O_5_ and (c) CeVO_4_ with Zn adsorption, and the corresponding *E*_b_.


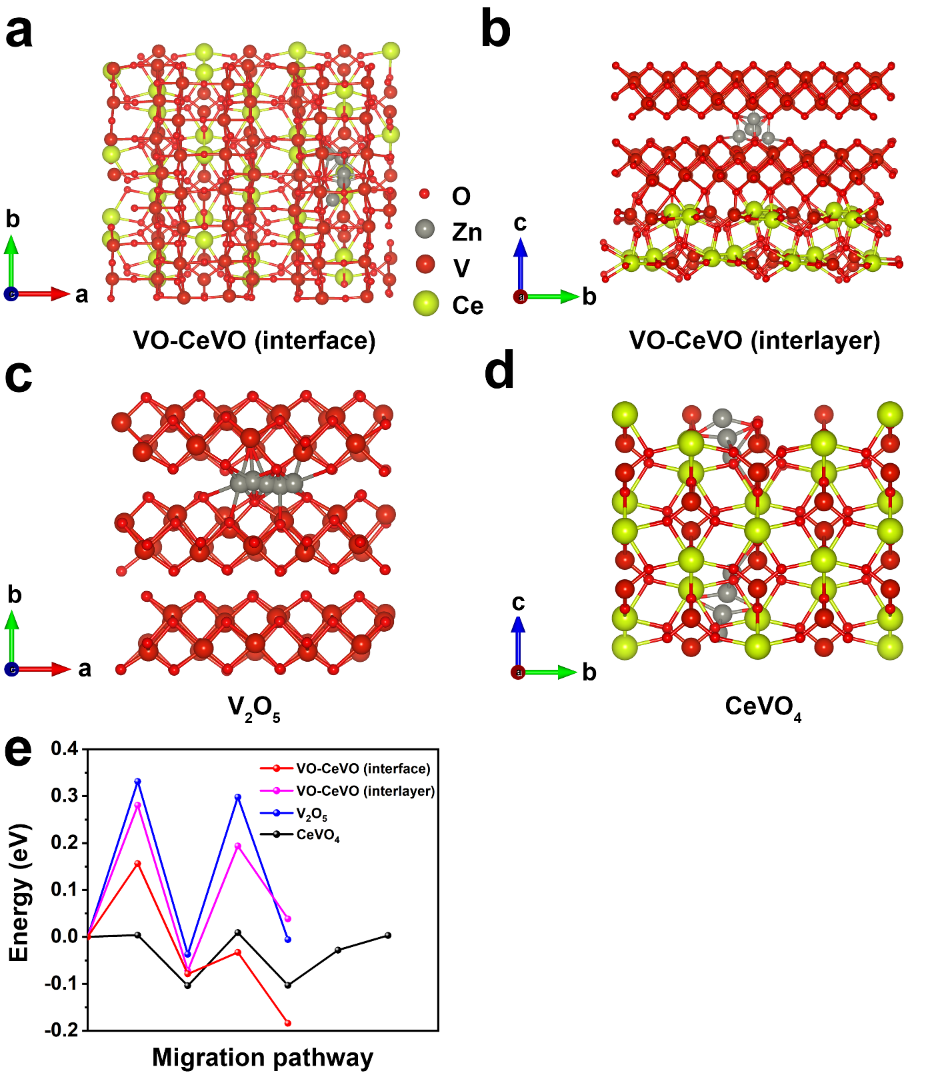


**Figure S22.** The Zn^2+^ diffusion paths on (a) VO-CeVO interface, (b) VO-CeVO interlayer, (c) V_2_O_5_ and (d) CeVO_4_ surface, and (e) corresponding energy barriers for Zn^2+^ diffusion.


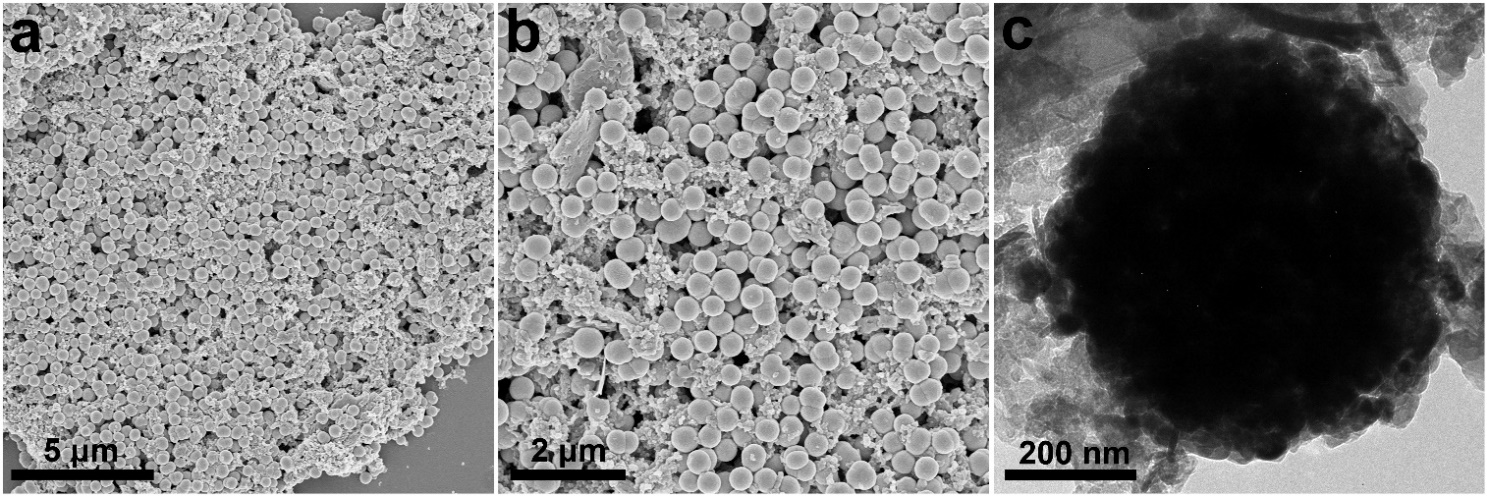


**Figure S23.** (a,b) FESEM and (c) TEM images of the VO-CeVO electrode at the fully discharged state.


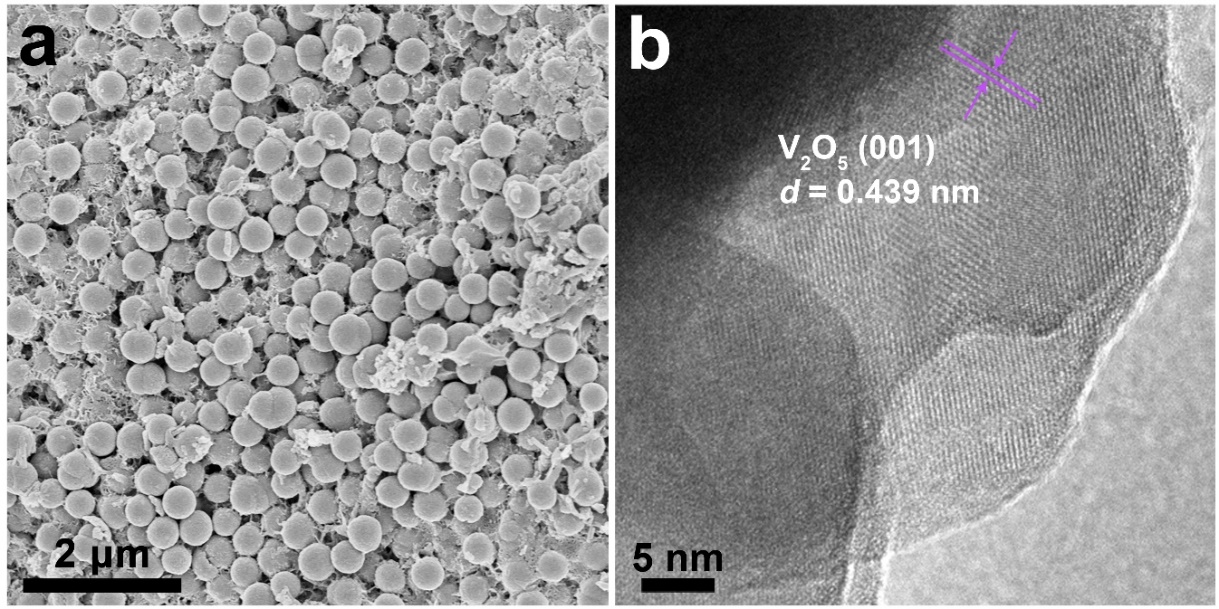


**Figure S24.** (a) FESEM and (b) HRTEM images of the VO-CeVO electrode at the fully charged state.


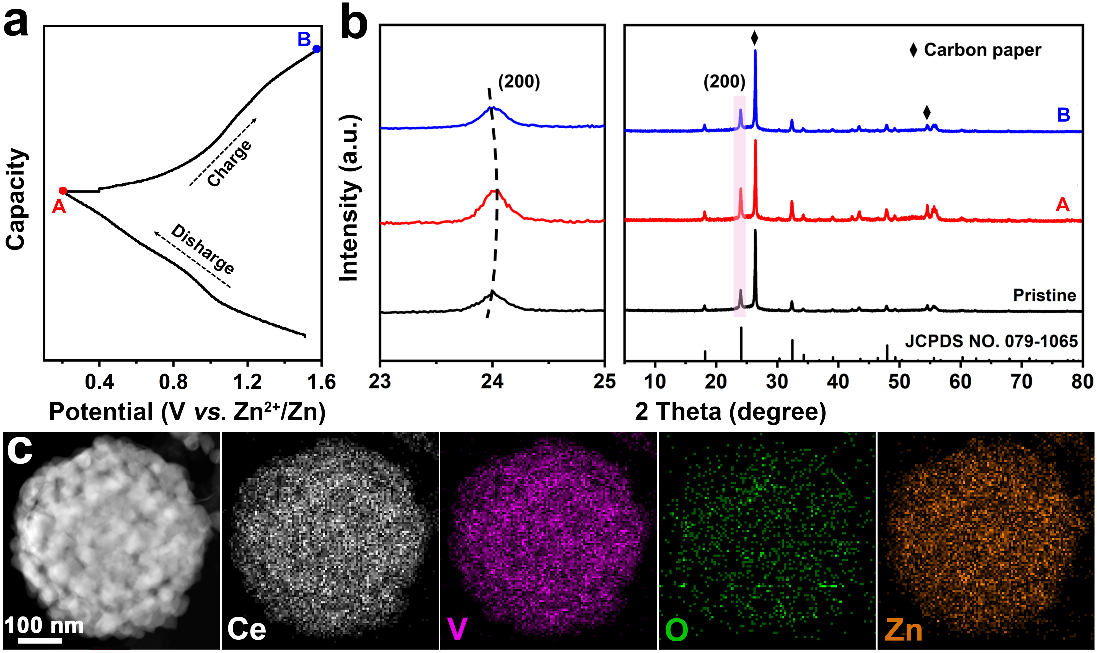


**Figure S25**. Structural evolution of the CeVO_4_ electrode during the discharge/charge processes. (a) GCD curve at 0.1 A g^-1^ and the corresponding ex situ (b) XRD patterns, (c) HAADF-STEM image and the corresponding elemental mapping images at the fully discharged state.


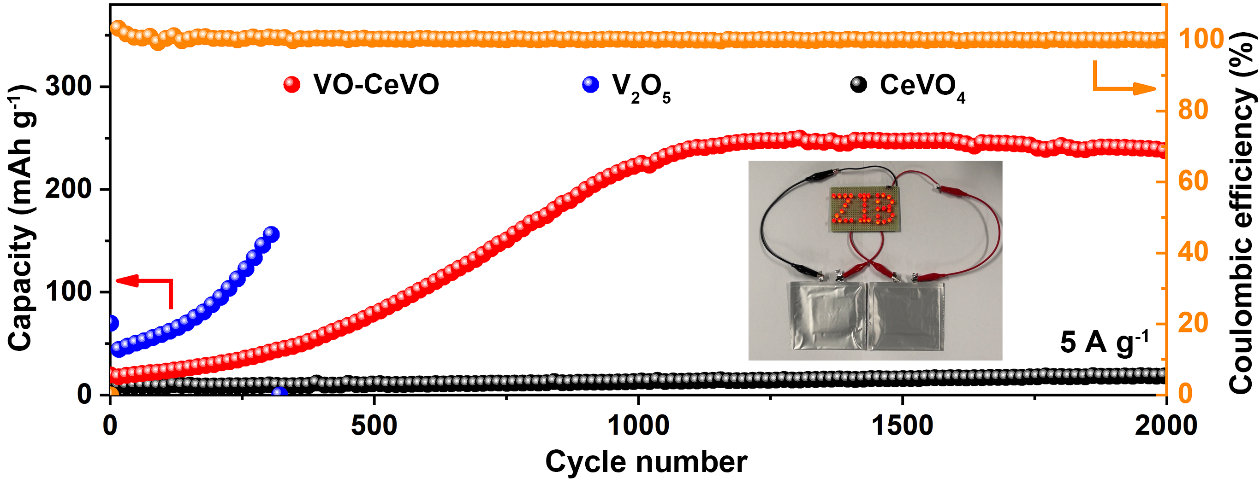


**Figure S26.** The cycling performance of Zn||VO-CeVO, Zn||V_2_O_5_ and Zn||CeVO_4_ pouch batteries at 5 A g^-1^ (inset: photograph of two connected Zn||VO-CeVO pouch batteries powering 36 bulbs with the ZIB logo).

**Table S1.** The contents of elements in NH_4_VO_3_-CeVO_4_ with different anion-exchange time.

| Samples | V (wt%) | Ce (wt%) | C (wt%) | N (wt%) |
| --- | --- | --- | --- | --- |
| NH_4_VO_3_-CeVO_4_-10 min | 42.5 | 7.4 | 4.2 | 4.9 |
| NH_4_VO_3_-CeVO_4_-60 min | 44.2 | 10.8 | 3.1 | 6.5 |
| NH_4_VO_3_-CeVO_4_-120 min | 50.6 | 11.6 | 3.0 | 6.7 |

**Table S2**. ICP-OES results of VO-CeVO.

| Elements | Content (wt%) |
| --- | --- |
| Ce | 14.1 |
| V | 48.4 |

**Table S3.** Comparison for the electrochemical performance of these representative V-based cathode materials for aqueous Zn-ion batteries.

| Cathode material | Voltage window | Rate performance | Cycling performance | Ref. |  |
| --- | --- | --- | --- | --- | --- |
| VO-CeVO | 0.2-1.6 V | 300 mAh g^-1^ at 1 A g^-1^,  257 mAh g^-1^ at 10 A g^-1^ | 193.1 mAh g^-1^ after 10000 cycles at 5 A g^-1^ | This work |  |
| NVO/PoPDA@GO | 0.2-1.6 V | 383 mAh g^-1^ at 1 A g^-1^,  180 mAh g^-1^ at 5 A g^-1^ | 167.8 mAh g^-1^ after 1000 cycles at 5 A g^-1^ | ^[7]^ |  |
| V_2_O_5_ | 0.2-1.6 V | 280.6 mAh g^-1^ at 0.5 A g^-1^,  128.6 mAh g^-1^ at 5 A g^-1^ | 213.2 mAh g^-1^ after 3500 cycles at 2 A g^-1^ | ^[8]^ |  |
| Ni_0.25_V_2_O_5_·nH_2_O | 0.3-1.7 V | 402 mAh g^-1^ at 0.2 A g^-1^,  147 mAh g^-1^ at 5 A g^-1^ | 218 mAh g^-1^ after 1200 cycles at 5 A g^-1^ | ^[9]^ |  |
| VS_2_/Ti_3_C_2_T_z_ | 0-2.0 V | 98.2 mAh g^-1^ at 0.2 A g^-1^,  92.2 mAh g^-1^ at 2 A g^-1^ | 89.2 mAh g^-1^ after 5000 cycles at 2 A g^-1^ | ^[10]^ |  |
| NaCa_0.6_V_6_O_16_·3H_2_O | 0.4-1.5 V | 347 mAh g^-1^ at 0.1 A g^-1^,  154 mAh g^-1^ at 5 A g^-1^ | 125 mAh g^-1^ after 10000 cycles at 5 A g^-1^ | ^[11]^ |  |
| CO_2_-V_6_O_13_ | 0.3-1.5 V | 341 mAh g^-1^ at 1 A g^-1^,  237 mAh g^-1^ at 5 A g^-1^ | 244 mAh g^-1^ after 4000 cycles at 2 A g^-1^ | ^[12]^ |  |
| PANI100-V_2_O_5_ | 0.4-1.4 V | 342 mAh g^-1^ at 1 A g^-1^,  216 mAh g^-1^ at 10 A g^-1^ | 208 mAh g^-1^ after 2000 cycles at 5 A g^-1^ | ^[13]^ |  |
| O-VN | 0.2-2.0 V | 673 mAh g^-1^ at 0.5 A g^-1^,  581 mAh g^-1^ at 3 A g^-1^ | 361 mAh g^-1^ after 200 cycles at 2 A g^-1^ | ^[14]^ |  |
| TMPA-VOH | | 0.2-1.6 V | 382 mAh g^-1^ at 1 A g^-1^,  326 mAh g^-1^ at 4 A g^-1^ | 162 mAh g^-1^ after 2000 cycles at 4 A g^-1^ | ^[15]^ |

NVO/PoPDA@GO: poly-o-phenylenediamine (PoPDA) intercalated into the interlayers of NH_4_V_3_O_8_ (NVO) and hybridized with graphene oxide (GO)

CO_2_-V_6_O_13_: V_6_O_13_ modified by CO_2_

PANI100-V_2_O_5_: polyaniline intercalated vanadium oxide

O-VN: oxygen-doped vanadium nitride

TMPA-VOH: [C_6_H_6_N(CH_3_)_3_]_1.08_V_8_O_20_·0.06H_2_O

**Supplementary References**

[1] X. Yang, Y. Hu, N. Dunlap, X. Wang, S. Huang, Z. Su, S. Sharma, Y. Jin, F. Huang, X. Wang, S. H. Lee, W. Zhang, *Angew. Chem. Int. Ed.* **2020**, *132*, 20565-20569.

[2] Z. Li, Y. Wei, Y. Liu, S. Yan, M. Wu, *Adv. Sci.* **2023**, *10*, 2206860.

[3] G. Kresse, J. Furthmüller, *Phys. Rev. B* **1996**, *54*, 11169-11186.

[4] J. P. Perdew, K. Burke, M. Ernzerhof, *Phys. Rev. Lett.* **1996**, *77*, 3865-3868.

[5] P. E. Blochl, *Phys. Rev. B* **1994**, *50*, 17953-17979.

[6] Y. J. Li, M. Wang, M. y. Tang, X. Tian, S. Gao, Z. He, Y. Li, T. G. Zhou, *Phys. Rev. B* **2016**, *75*, 169-173.

[7] M. Li, M. Liu, Y. Lu, G. Zhang, Y. Zhang, Z. Li, Q. Xu, H. Liu, Y. Wang, *Adv. Funct. Mater.* **2024**, *34*, 2312789.

[8] L. Zhou, P. Li, C. Zeng, A. Yi, J. Xie, F. Wang, D. Zheng, Q. Liu, X. Lu, *Adv. Sci.* **2024**, *11*, 2305749.

[9] J. Li, K. McColl, X. Lu, S. Sathasivam, H. Dong, L. Kang, Z. Li, S. Zhao, A. G. Kafizas, R. Wang, D. J. L. Brett, P. R. Shearing, F. Corà, G. He, C. J. Carmalt, I. P. Parkin, *Adv. Energy Mater.* **2020**, *10*, 2000058.

[10] L. Zhang, Y. Li, X. Liu, R. Yang, J. Qiu, J. Xu, B. Lu, J. Rosen, L. Qin, J. Jiang, *Adv. Sci.* **2024**, *11*, 2401252.

[11] K. Zhu, T. Wu, K. Huang, *Adv. Energy Mater.* **2019**, *9*, 1901968.

[12] W. Shi, B. Yin, Y. Yang, M. B. Sullivan, J. Wang, Y. W. Zhang, Z. G. Yu, W. S. V. Lee, J. Xue, *ACS Nano* **2021**, *15*, 1273-1281.

[13] S. Chen, K. Li, K. S. Hui, J. Zhang, *Adv. Funct. Mater.* **2020**, *30*, 2003890.

[14] D. Chen, M. Lu, B. Wang, R. Chai, L. Li, D. Cai, H. Yang, B. Liu, Y. Zhang, W. Han, *Energy Storage Mater.* **2021**, *35*, 679-686.

[15] X. Jia, C. Liu, Z. Wang, D. Huang, G. Cao, *Nano-Micro Lett.* **2024**, *16*, 129.
